# Functional network topography of the medial entorhinal cortex

**DOI:** 10.1073/pnas.2121655119

**Published:** 2022-02-08

**Authors:** Horst A. Obenhaus, Weijian Zong, R. Irene Jacobsen, Tobias Rose, Flavio Donato, Liangyi Chen, Heping Cheng, Tobias Bonhoeffer, May-Britt Moser, Edvard I. Moser

**Affiliations:** ^a^Kavli Institute for Systems Neuroscience, Norwegian University of Science and Technology (NTNU), Trondheim 7030, Norway;; ^b^Centre for Neural Computation, NTNU, Trondheim 7030, Norway;; ^c^Institute for Experimental Epileptology and Cognition Research, University of Bonn, Bonn 53127, Germany;; ^d^Biozentrum, University of Basel, Basel 4056, Switzerland;; ^e^State Key Laboratory of Membrane Biology, Institute of Molecular Medicine, Peking-Tsinghua Center for Life Sciences, College of Future Technology, Peking University, Beijing 100871, China;; ^f^PKU-IDG/McGovern Institute for Brain Research, Beijing 100871, China;; ^g^Beijing Academy of Artificial Intelligence, Beijing 100101, China;; ^h^Research Unit of Mitochondria in Brain Diseases, Chinese Academy of Medical Sciences, PKU-Nanjing Institute of Translational Medicine, Nanjing 211899, China;; ^i^Synapses–Circuit–Plasticity, Max Planck Institute of Neurobiology, Planegg-Martinsried 82152, Germany

**Keywords:** entorhinal cortex, spatial coding, topography, two-photon microscopy, grid cells

## Abstract

The medial entorhinal cortex (MEC) creates a map of local space, based on the firing patterns of grid, head-direction (HD), border, and object-vector (OV) cells. How these cell types are organized anatomically is debated. In-depth analysis of this question requires collection of precise anatomical and activity data across large populations of neurons during unrestrained behavior, which neither electrophysiological nor previous imaging methods fully afford. Here, we examined the topographic arrangement of spatially modulated neurons in the superficial layers of MEC and adjacent parasubiculum using miniaturized, portable two-photon microscopes, which allow mice to roam freely in open fields. Grid cells exhibited low levels of co-occurrence with OV cells and clustered anatomically, while border, HD, and OV cells tended to intermingle. These data suggest that grid cell networks might be largely distinct from those of border, HD, and OV cells and that grid cells exhibit strong coupling among themselves but weaker links to other cell types.

## Significance

The investigation of the topographic organization of spatially coding cell types in the medial entorhinal cortex (MEC) has so far been held back by the lack of appropriate tools that enable the precise recording of both the anatomical location and activity of large populations of cells while animals forage in open environments. In this study, we use the newest generation of head-mounted, miniaturized two-photon microscopes to image grid, head-direction, border, as well as object-vector cells in MEC and neighboring parasubiculum within the same animals. The majority of cell types were intermingled, but grid and object-vector cells exhibited little overlap. The results have implications for network models of spatial coding.

The topographic organization of functional cell types in cortical areas has long been recognized as one of the fundamental organizational principles in the vertebrate brain (1) (for a review, see ref. 2). In mammals, the large-scale, anatomical arrangement of function is most apparent in primary sensory cortices. These regions frequently possess an internal, large-scale, topographic organization with respect to the stimulus space they cover, leading to the emergence of, for example, orientation maps, ocular dominance maps, and retinotopic maps in primary visual cortex (3, 4) or tonotopic maps in primary auditory cortex (5, 6).

While these macroscale patterns are relatively conserved across mammals, the extent to which single-cell tuning properties are topographically organized within each brain region varies from species to species (7, 8). The underlying principles that dictate such patterning may derive from the constraints that speed and energy of synaptic communication impose on brain networks: Cell types within a network that depend on frequent and dense information exchange may benefit from more direct anatomical proximity to each other than those that require less interaction (9–12), similar to how computational efficiency of parallel computing networks is increased if communication overhead is minimized (13). This is particularly true in brain regions in which circuit architecture might be less controlled by extrinsic factors than in many primary sensory cortices, and where the architecture instead follows more intrinsic organizational principles. In such higher-order regions, the study of functional topography can shape ideas about network topologies that determine both the local connectivity of cells as well as the emergence and maintenance of their tuning properties. One of the best illustrations of this concept in nonmammalian species is the discovery of a ring-shaped head-direction (HD) circuit in the centroid complex of fruit flies (14–16), whose anatomical layout and wiring mirror the predictions of ring attractor network models for directional tuning (17–19). The anatomical layout of the fruit fly HD system minimizes the wiring distance between similarly tuned dendrites.

In the mammalian brain, higher association cortices may follow similar principles. One such region where topographic organization may benefit computation is the mammalian medial entorhinal cortex (MEC), which contains a variety of distinct but functionally correlated cell types that together form a map of local space. These are grid cells (20), whose firing fields tessellate the environment in equilateral triangles; border cells, which fire close to environmental boundaries (21–23); object-vector (OV) cells, which are active in fixed direction and distance from objects (24); and HD cells, that are active when the animal points its head toward specific directions (25, 26). The abundance of these well-characterized cell types makes MEC an ideal candidate for the study of functional network topography, which in turn can lend evidence to the validity of network models describing the emergence of spatial coding in this region. Prominent models of HD and grid network topologies, so-called continuous attractor network (CAN) models, predict a dense, functional connectivity within networks of the same cell type, which in turn suggests that they should cluster anatomically [HD cells (17–19); grid cells (27–31)].

To elucidate these organizational principles, anatomically precise location and activity data are required. Two-photon (2P) in vivo calcium imaging is ideally suited for this because of its optical sectioning capability, high resolution, and ability to record from hundreds of cells simultaneously over large fields of view (FOVs) (32–34). Using large-scale 2P in vivo calcium imaging in head-fixed animals, recent studies have reported clustering of grid cells and associated tuning properties within MEC (35, 36), supporting attractor network models that predict such patterns. However, due to the constraints imposed by the experimental setup, the analysis of other spatially modulated cell types was either impeded or rendered impossible (e.g., for HD cells) (37). While macroscopic gradients have been described in freely moving animals for both grid cells (20, 38–41) and HD cells (42), a description of both the macro- as well as microscale anatomical organization of all known, spatially modulated cell classes in MEC and adjacent brain regions is still lacking.

Here, we show results from imaging large areas of layers II/III of MEC and neighboring structures in mice using an upgraded version of the latest published miniaturized, portable two-photon microscopes (2P miniscopes) (43, 44). The modification to the previously published design enabled us to record from more than a hundred neurons simultaneously and resolve their spatial tuning properties at the same time as animals foraged freely in square open-field arenas. The data enabled us to obtain a detailed record of anatomical organization within and between functional cell classes in the brain under quasi-natural conditions.

We acquired stable, long-term, dual-channel recordings through chronic implants consisting of either a gradient refractive index (GRIN) lens combined with a microprism or a prism only (45), in both cases positioned between MEC and cerebellum. With data from these recordings, we show that grid cells cover anatomically stable territories across superficial layers of MEC and neighboring regions that are distinct from those of other functional cell types, most notably OV cells, which showed low co-occurrence with grid cells. These effects were maintained when we included conjunctive cells (i.e., those that crossed cutoff criteria for more than one cell class). Overall, all cell classes except for grid cells intermingle. Grid cells, but not other cell types, appear to cluster anatomically. The sharpest transitions between functional territories were visible at the border of MEC and neighboring parasubiculum (PAS).

These observations point to a unique topographic organization of grid and other spatial cell types in MEC. Grid cells seem to exist in relative isolation from other spatially modulated cells and may thereby form subnetworks that are relatively shielded from input of other cell classes.

## Results

### Large-FOV Recordings with 2P Miniscopes in Freely Moving Mice.

**Fig. 1.**
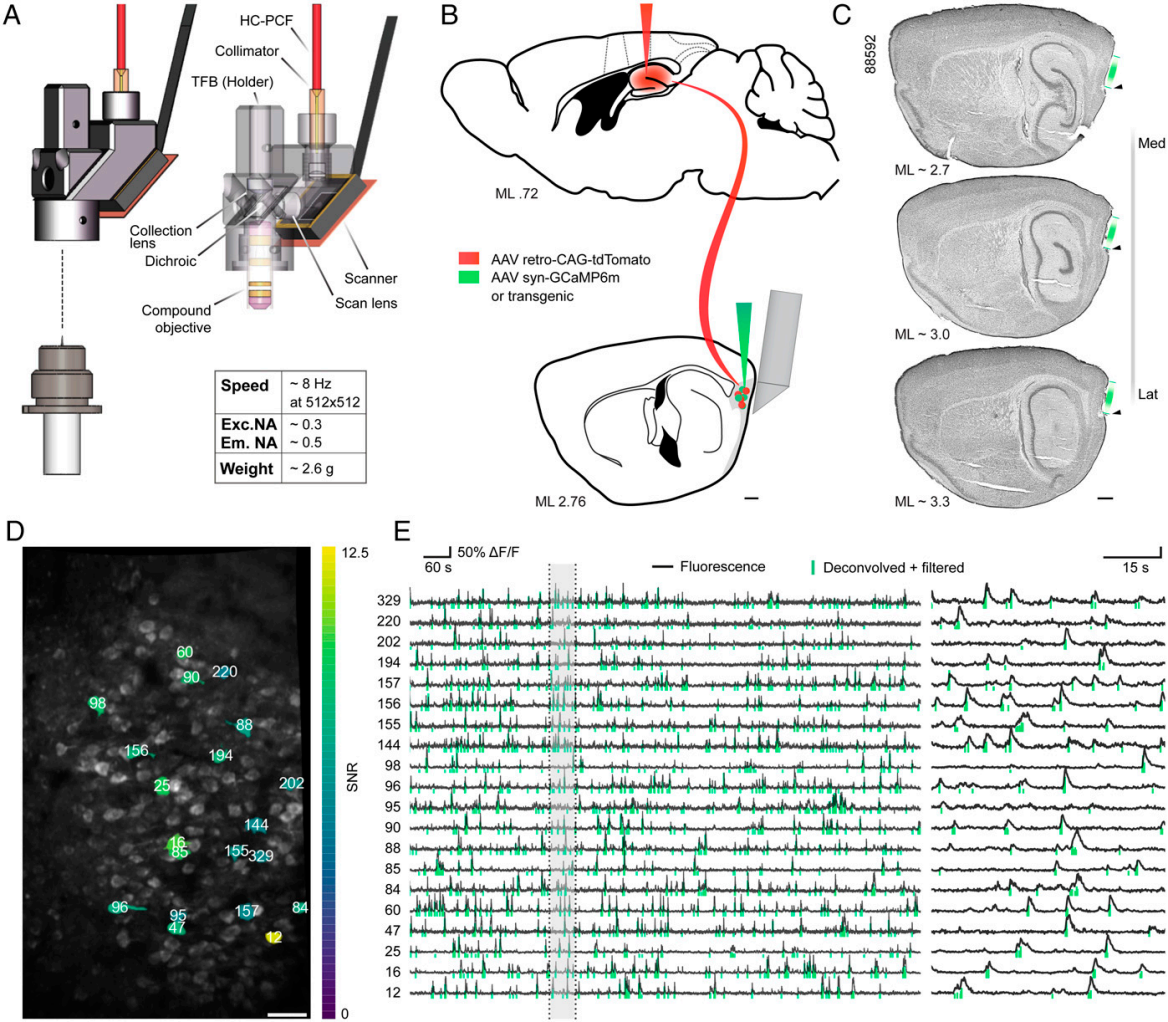
Calcium imaging with the 2P miniscope in MEC. (*A*) Schematic of the miniscope. Table shows speed, numerical aperture (NA) specifications, and weight. Em. NA, emission NA; Exc. NA, excitation NA; HC-PCF, hollow-core photonic crystal fiber; TFB, tapered fiber bundle. (*B*) Sagittal brain section schematics at two medio-lateral (ML) positions. Green, GCaMP expression; red, retro-AAV expressing tdTomato. (Scale bar, 500 µm.) (*C*) Nissl-stained, sagittal brain slices from one animal. The approximate medio-lateral (ML) position is indicated. Green lines show the region accessible through GRIN + prism. The ventral implant border is indicated by black arrowheads. Mouse ID is indicated (*Top Left*). (Scale bar, 500 µm.) (*D*) Example maximum-intensity projection of one open-field session with a total of 213 detected cells (after SNR filtering). Colored regions show a subset of extracted cells. Numbers indicate Suite2p cell IDs. (Scale bar, 50 µm.) (*E*) Fluorescent (normalized Δ*F*/*F*, black traces) and deconvolved and filtered signal (green vertical bars) of 20 example cells (colored ROIs in *D*). Total session length, ∼1,200 s. The shaded region (60-s excerpt) is magnified (*Right*).

To obtain the anatomical and functional data required for topographic analyses of large numbers of spatially modulated cells in MEC, we used a fundamentally updated version of a previously published 2P miniscope (44), sharing decisive features with an even further developed version of the miniscope (“MINI2P”) (46) (Fig. 1*A* and *SI Appendix*, Fig. S1*A*). The present miniscope features a sufficiently large FOV for neural population recordings (width × height with GRIN + prism after motion correction [mean ± SD], 367 ± 40 × 558 ± 50 µm; *SI Appendix*, Fig. S1*B*) as well as sufficiently low weight (2.6 g without tether) to enable prolonged and unrestrained recordings in freely moving mice (*SI Appendix*, Fig. S1*A*). The weight decrease of this modified miniscope compared with its predecessors was achieved in both the scope itself, now made from a type of machinable plastic, as well as in the connection cable, which now contains a much thinner, tapered fiber bundle with decreased overall diameter (46). While lowering the weight, these design changes also drastically increased the degree to which the head-mounted assembly could be twisted and thereby permitted animals to exhibit more naturalistic foraging behaviors (46). Some of the newest hardware features of MINI2P that are introduced in ref. 46, such as the even further enlarged FOV size, the built-in Z-focusing, and the ability to shift FOVs systematically between sessions, were included in only a small subset of the present data (18 out of 212 recordings); importantly, however, all present recordings had the lowered weight and the thinner, more flexible connection cable of MINI2P, allowing for good spatial coverage and reliable classification of spatially modulated cell types (see below).

### Chronic Imaging of MEC and Adjacent Structures.

Imaging was either performed in transgenic animals that expressed GCaMP6s ubiquitously in excitatory cells (Camk2a-tTA x tetO-G6s, “transgenic”) or in wild-type mice that had been injected with recombinant adeno-associated viruses (AAVs) expressing GCaMP6m (“virus,” synapsin promoter) (Fig. 1*B*). Both indicators are highly sensitive and yield comparable fractions of responsive cells in visual cortex experiments (47). Moreover, the relatively slow scanning rate of the miniscope Micro-Electro-Mechanical Systems (MEMS) scanner (∼7.52 fps) made GCaMP6s and GCaMP6m preferable over faster indicators. To gain access to the superficial layers of MEC and neighboring brain regions, we chronically implanted optical relays consisting of a GRIN and prism doublet (diameter 1 mm, overall length ∼4.7 mm; Fig. 1 *B*, *Bottom* and *SI Appendix*, Fig. S1 *B*, *Right*) or prisms only (1.3 × 1.3 × 1.6 mm) in-between cerebellum and MEC, such that the prism surface was centered on and flush against the superficial layers of MEC (Fig. 1 *B* and *C*). This approach enabled us to image MEC chronically for weeks and made it possible to capture the activity of over a hundred cells simultaneously after filtering for signal-to-noise ratio (SNR) (example in Fig. 1 *D* and *E*) (SNR across 24,820 cells, virus [median] 5.6, transgenic 4.8; *SI Appendix*, Fig. S1*C*; number of cells across *n* = 133 sessions after filtering, virus [mean ± SD] 112 ± 68, transgenic 161 ± 96; *SI Appendix*, Fig. S1*D*). To improve anatomical specificity in vivo, we labeled superficial layer MEC cells selectively by injecting retrogradely transported AAVs carrying tdTomato into ipsilateral hippocampus (Fig. 1*B*) in most animals (13 out of 15). This resulted in labeling of mostly layer III and some layer II cells in MEC (*SI Appendix*, Fig. S1 *E–G*) and thereby allowed us to distinguish MEC from neighboring structures like the medially located PAS as well as layer II from layer III by expression differences (i.e., abundance of tdTomato cell labeling in layer III compared with layer II).

### 2P Miniscope Imaging Enables Analysis of All Spatial Cell Types.

**Fig. 2.**
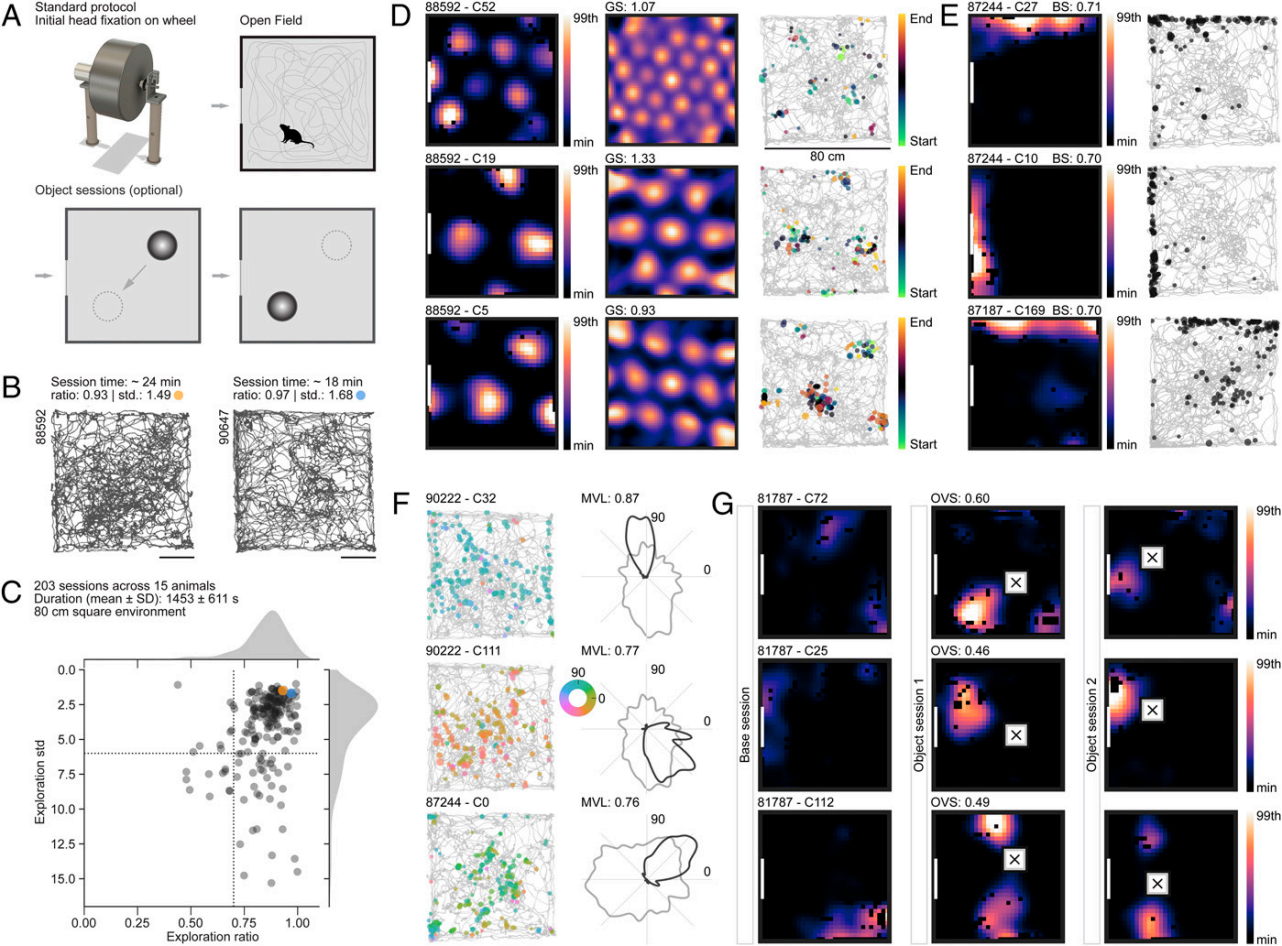
2P miniscope imaging of all functional cell types in MEC during unrestrained behavior. (*A*) Schematic of behavioral protocol. Initial head fixation on a wheel and foraging in a square open-field arena (“Standard protocol”). When screening for OV cells [“Object sessions (optional)”], we added either one object which was moved between two object sessions, or two objects in a single session. (*B*) Example path plots (animal number is indicated *Top Left*). Session time, exploration ratio, and exploration SD are indicated. Colored dots (orange and blue) are referenced in *C*. (Scale bars, 20 cm.) (*C*) Analysis of coverage (occupancy) of the open-field environment for 203 sessions recorded in 15 animals (80 × 80 cm^2^ open-field arena, duration 1,453 ± 610.6 s, mean ± SD). Plotted is the exploration ratio (“ratio”) versus exploration SD (“std”). Stippled lines show arbitrary cutoffs, splitting the data into quadrants of “better” (*Top Right*) and “worse” coverage (*Bottom Left*). Sessions shown in *B* are indicated in orange and blue. (*D–G*) Example grid (*D*), border (*E*), HD (*F*), and OV (*G*) cells recorded during ∼20 min of free foraging in an 80 × 80 cm open field. (*D*) Grid cells. (*D*, *Left*) Spatial tuning maps for grid cells of a module with short grid spacing (*Top*) and two co-recorded grid cells from a module with larger spacing (*Middle* and *Bottom*). White lines indicate the position of a (single) cue card, fixed to the wall of the box. (*D*, *Center*) Two-dimensional autocorrelations of spatial tuning maps on the *Left* (GS, grid score). (*D*, *Right*) Path and deconvolved signal (amplitude is indicated by dot size, and session time is indicated by color). (*E*) Border cells. (*E*, *Left*) Spatial tuning maps of border cells with border score (BS) indicated above the spatial tuning map. The first two cells (animal 87244, cells 27 and 10) were co-recorded. (*E*, *Right*) Path and deconvolved signal (amplitude is indicated by dot size). (*F*) HD cells. Path and deconvolved signal (*Left*) and tuning curves (*Right*) of three example HD cells. Tuning strength (mean vector length; MVL) is displayed above the tuning curve. Deconvolved signal is shown color-coded by HD on top of the path plot. Tuning curves show normalized occupancy (gray line) and the cell’s directional tuning curve (black line). (*G*) OV cells. Spatial tuning maps of three OV cells, two with one field (*Top* and *Middle*), and one with two fields (*Bottom*). White squares with a black cross indicate the position of the object. OV score (OVS) is displayed above the *Center* tuning map. Color maps have the same limits across all sessions for one cell (range of baseline session).

Spatially modulated cell types have classically been studied in animals that were allowed to engage in unrestrained foraging behavior in open-field arenas. Due to the low weight of the 2P miniscope and its thin and flexible connection cable, we were likewise able to let animals run in large square boxes over tens of minutes (Fig. 2*A*). Open-field arenas were mostly 80 × 80 cm in size, although a few sessions were run in 60 × 60 and 100 × 100 cm arenas. Animals readily engaged in natural exploratory behaviors in all of these arenas while foraging for cookie crumbs (Fig. 2 *A* and *B* and *SI Appendix*, Fig. S2*A*). Optimal coverage was achieved after just ∼20 min (Fig. 2 *B* and *C*; 15 animals, 203 sessions), which greatly facilitated the interpretation of spatial modulation in recorded cells. Average and maximum speed of animals during recordings matched that of previous reports using chronic implants in mice [for example, chronic implantations of tetrode bundles for electrophysiological recordings (48)] (*SI Appendix*, Fig. S2*B*). Moreover, the behavior stayed stable over long durations such that we could record multiple sessions in a row (*SI Appendix*, Fig. S2*C*) and animals required only a few resting periods when inside the open-field arena (*SI Appendix*, Fig. S2*D*). This was of pivotal importance for the analysis of OV cells, which are analyzed by running consecutive sessions (usually one baseline session without objects and two object sessions) to follow the emergence of object-related firing fields and their reallocation when the object(s) is moved between sessions (24).

In this way, we were able to analyze all major spatial classes, namely grid (Fig. 2*D*), border (Fig. 2*E*), HD (Fig. 2*F*), and OV (Fig. 2*G*) cells. To assign cells to a given cell type (i.e., grid, HD, border, or OV), we compared their spatial or directional tuning scores (e.g., grid score for grid cells) with shuffled distributions created by shifting the tracking and fluorescence signal for every cell against each other in random time intervals (*SI Appendix*, *Extended Methods*). For grid, border, and HD cells, we used single criteria with 95th percentile shuffling cutoffs, if not otherwise indicated, and combined criteria were used to classify OV cells (*SI Appendix*, *Extended Methods*). Scores matched those of previous reports using electrophysiological methods (49). Due to the high SNR of the recorded signal and precision increases achieved by deconvolving the fluorescent traces (50), we were able to resolve closely spaced firing fields of grid cells (see example in Fig. 2 *D*, *Top*) as well as precise HD tuning (see example in Fig. 2 *F*, *Top*), despite the slow time courses of calcium dynamics. Cells that were recorded across adjacent recording sessions exhibited reproducible, stable tuning (measured both for HD modulation as well as spatial tuning map correlation; *SI Appendix*, Fig. S2 *E* and *F*). Due to the high throughput achievable in our large-scale imaging approach, we were able to determine the properties of tens of OV cells simultaneously while obtaining field-object distance and angle distributions that closely resemble those of previously published reports (24) (*SI Appendix*, Fig. S2*G*).

Our imaging approach thus enabled high-throughput identification of spatial cell types and anatomical positions of over a hundred simultaneously recorded cells in MEC and adjacent structures.

### OV Cells and Grid Cells Segregate.

**Fig. 3.**
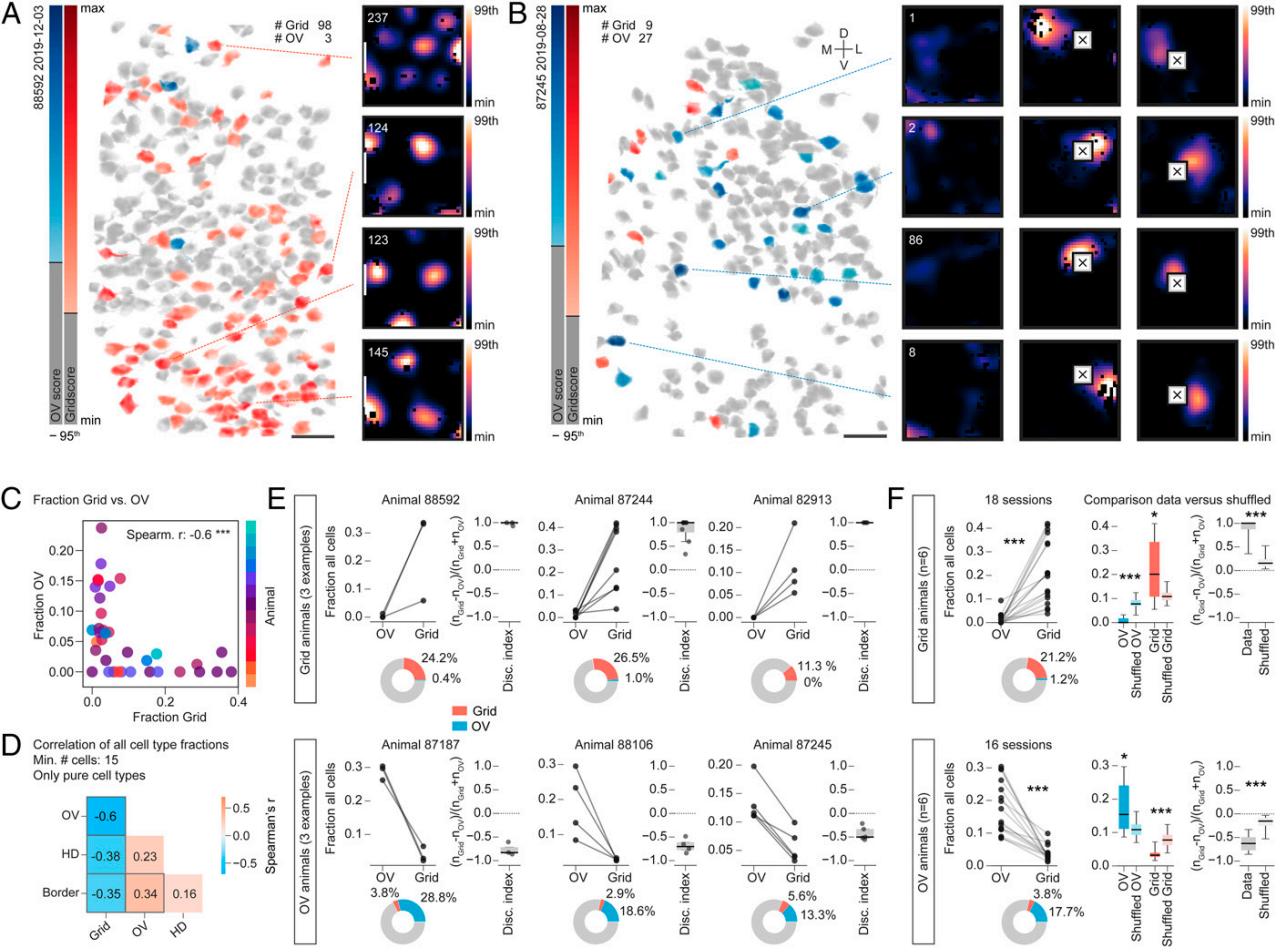
Grid cells and OV cells occupy discrete regions of MEC. (*A* and *B*) Anatomical distribution of grid and OV cells and spatial tuning map examples in two different animals (animal name and recording date are at *Top Left*). Cells are color-coded by tuning strength (OV score, blue; grid score, red). Cells not meeting cutoff criteria are shown in gray. (Scale bars, 50 µm.) (*A*) Recording with 98 grid cells and 3 OV cells. (*B*) Recording with 27 OV cells and 9 grid cells. Tissue orientation is indicated by a cross (D, dorsal; L, lateral; M, medial; V, ventral). (*C*) Fraction of (pure) grid versus OV cells in 36 sessions across 13 animals. Each dot represents a session and animals are coded by color. Spearman’s correlation is shown (*Top Right*) (Spearman’s *r* = −0.596, *n*_Grid_ = *n*_OV_ = 36; ***P = 0.00013). (*D*) Summary of correlations as in *C*, but for all cell types. Spearman’s *r* is color-coded and shown as text in the center of each box (filtered by MEC, minimum cutoff of 15 cells after filtering, only pure cell types). A thick gray border around some squares indicates significance (*P* < 0.05) in Spearman’s correlation result. See *SI Appendix*, *Extended Results* for detailed statistics. (*E*) Fraction of (pure) grid and OV cells and the discrimination index over grid and OV cells in each session across six example animals (minimum cutoff of 15 cells), with higher fractions of grid cells than OV cells (*Top*) or the opposite (*Bottom*). Pie charts show the average percentage of grid and OV cells across sessions. Boxes in boxplots extend from lower to upper quartiles of the data; the horizontal line indicates population median; whiskers indicate 1st to 99th percentile; dashed line indicates 0. (*F*, *Left*) Summary across all sessions and animals shown in *E*. Grid: six animals, Mann–Whitney *U* = 320, *n*_Grid_ = *n*_OV_ = 18, ***P = 3.82e-07, two-sided; OV: six animals, Mann–Whitney *U* = 3, *n*_Grid_ = *n*_OV_ = 16, ***P = 2.70e-06, two-sided. (*F*, *Right*) Fraction of cell types (OV or grid) and discrimination indices in comparison with a shuffled distribution. Asterisks indicate results of two-sided Wilcoxon signed-rank tests of the data versus the population mean of the shuffled data (*P < 0.05, **P < 0.01, ***P < 0.001). Boxplot properties are as in *E*. See *SI Appendix*, *Extended Results* for detailed statistics.

While imaging in MEC and neighboring regions, we noticed that recordings that showed an abundance of grid cells in one FOV (∼20% of all imaged cells) often would have comparably few OV cells (see example in Fig. 3*A*, 98 grid cells versus 3 OV cells) and, conversely, recordings with multiple OV cells would have only few co-recorded grid cells (see example in Fig. 3*B*, 27 OV cells versus 9 grid cells). Across all recordings, this divergence was most visible in animals in which 10% or more of either cell type were recorded on average (Fig. 3*C* and *SI Appendix*, Fig. S2*H*). While differences in cell-type composition were visible between other pairs of spatially modulated cell classes as well (Fig. 3*D* and *SI Appendix*, Fig. S3 *A* and *B*), the most striking difference in cell numbers was found for grid versus OV cells (Fig. 3 *C* and *D*). This difference was maintained when we included conjunctive cells (i.e., those that crossed cutoff criteria for more than one cell class) (*SI Appendix*, Fig. S3*A*).

We subsequently identified 12 out of 15 animals for which object sessions had been run and where on average 10% of cells or more crossed grid or OV cell selection criteria (*SI Appendix*, Fig. S2*H*). Depending on whether the fraction of grid or OV cells was higher, these animals were classified as either “Grid” (Fig. 3 *E*, *Top*; three example animals) or “OV” animals (Fig. 3 *E*, *Bottom*; three example animals). In these two groups of animals (Grid and OV), grid and OV cells maintained stable anticorrelated relationships on the population level across multiple sessions (Fig. 3*E*, fraction of OV and grid cells in relation to all cells; six additional animals are shown in *SI Appendix*, Fig. S3*C*). In grid animals, the mean percentage of grid cells (±SD) was 21.2 ± 13.0%, whereas OV cells accounted for only 1.2 ± 2.3%; in OV animals, the percentage of grid cells was 3.8 ± 2.2%, as compared with 17.7 ± 7.8% for OV cells (mean ± SD) (Fig. 3 *F*, *Left*). To determine the size of the bias in absolute numbers of grid and OV cells, we calculated a discrimination index for each recording (“Disc. Index,” (*n*_Grid_ − *n*_OV_)/(*n*_Grid_ + *n*_OV_); Fig. 3 *E* and *F*), ranging from −1 (no grid cells, only OV cells) to 1 (only grid cells). To assess whether the differences in cell fractions and discrimination indices were bigger than expected by chance, we created a shuffled distribution, by labeling a subset of all cells as either grid or OV cells, and matching the observed combined frequency of grid and OV cells in our data (932 out of 4,399 cells across 34 sessions). We chose a ratio of 50% grid and 50% OV cells, mimicking a balanced population ratio, and drew random subsets from the total cell population that matched the average population size of our recorded sessions (*n* = 129 cells per sample). We then classified these shuffled sets as either OV or grid, depending on which cell type was represented more often, as we did for the recorded data. We observed that the differences in percentages of grid and OV cells in the data were more striking than in the shuffled set, both for shuffled Grid (Fig. 3 *F*, *Right Top*; mean ± SD, shuffled grid 11.1 ± 2.1%, shuffled OV 7.8 ± 1.9%, *n* = 4,591 shuffles) as well as shuffled OV (Fig. 3 *F*, *Right Bottom*; shuffled grid 7.83 ± 1.9%, shuffled OV 11.1 ± 2.2%, *n* = 4,558 shuffles). A similar effect was present in the discrimination indices. Compared with the shuffled distribution, which showed indices close to 0 for shuffled grid and OV sets (Fig. 3 *F*, *Right*; mean ± SD, grid 0.175 ± 0.118, OV −0.177 ± 0.119), the indices in our data were shifted strongly toward 1 in Grid animals and −1 in OV animals (Fig. 3 *F*, *Right*; mean ± SD, grid 0.879 ± 0.217, OV −0.617 ± 0.187), indicating substantially stronger biases than expected by chance. The effects were maintained when we relaxed criteria for OV cell classification, either by maintaining only a subset of the usually employed cutoff criteria or by dropping all but one (the 95th percentile of OV score shuffling distribution) (*SI Appendix*, Fig. S3*D*).

The observed stability of the bias toward either cell type is striking, considering that we sampled from varying depths and thus slightly different populations of cells in MEC layer II across days. Most of our recordings were located superficial to layer III, within which the majority of tdTomato-expressing cells were found (see *SI Appendix*, Figs. S1 *E–G* and S4 for example histology and red cell quantification in one Grid [*Top*] and one OV [*Bottom*] animal). Due to the large area covered by our implants and hence the difficulty to precisely align postmortem histology and in vivo imaging results, we were not able to further pinpoint the exact anatomical location of our imaging FOVs within or around MEC. We noticed, however, that grid animals were on average implanted close to the PAS border, while OV animals were implanted either more medially or more laterally than grid animals (all implant positions in *SI Appendix*, Fig. S2*H*, and forthcoming analysis).

In summary, we observed that grid cells show a low probability of co-occurrence with OV cells. This trend was maintained across recording days and animals.

### Anatomical Separation of Grid Cells from Other Functional Cell Types in MEC.

**Fig. 4.**
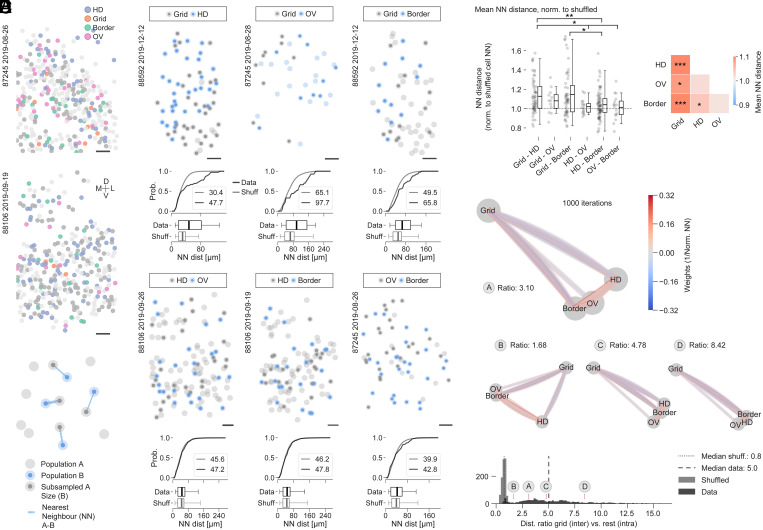
Anatomical separation of grid cells from other functional cell types in MEC. (*A*) Example cell maps of two recordings (OV animals 87245 [322 cells] and 88106 [309 cells]), showing the distribution of HD (blue), grid (orange), border (green), and OV (pink) cells. Cells are color-labeled if they crossed shuffling cutoff criteria for not more than one cell class. All other corecorded cells are shown in light gray if they crossed none or dark gray if they crossed more than one cutoff criterion. (Scale bars, 50 µm.) (*B*) Schematic of the NN analyses shown in this figure. The NN distances were extracted from size-matched (subsampled) populations of starter cells. Lines indicate NN (A to B) distances (line thickness indicates distance; thinner = longer). (*C*) Representative cell maps and NN distance analyses for six sample pairwise comparisons across multiple animals. Graphs below cell maps show the cumulative distribution and boxplots over NN distances (black) in comparison with a shuffled distribution (gray). Numbers in *Insets* indicate population averages. Minimum number of starter cells is five; minimum cell center distance is 10 µm. Boxes extend from lower to upper quartile values of the data, with a vertical line indicating the population average; whiskers indicate 1st to 99th percentile. (Scale bars, 50 µm.) (*D*) Normalized mean NN distances over all sessions and animals. (*D*, *Left*) Scatterplot showing one dot per dataset (95th percentile shuffling cutoffs were used throughout). Statistics above indicate results of Mann–Whitney *U* test (*P < 0.05, **P < 0.01). (*D*, *Right*) Color-coded average of results on the *Left*. Asterisks indicate results of two-sided one-sample *t* test against a population mean of 1 (*P < 0.05, **P < 0.01, ***P < 0.001); ns, not significant (*P* > 0.05). See *SI Appendix*, *Extended Results* for detailed statistics. (*E*) Spring-loaded network model; each node represents one cell class. Edges in the graph are color-coded by weight (1/normalized NN distance). (*E*, *Top*) Graph shows an example result of one simulation after 1,000 simulation steps. (*E*, *Middle*) More examples with increasing ratios. (*E*, *Bottom*) Results of graph distance ratios after shuffling weights (1,000 permutations) versus data (shuffled: lighter gray, median ratio 0.8 [small dashed line in histogram]; data: darker gray, median ratio 5.0 [large dashed line in histogram]). Ratios of the example graphs shown above (graphs *A–D*) are indicated.

We observed that the fractions of grid and other cell types, particularly OV cells, frequently appeared anticorrelated on the session level (Fig. 3). We therefore wondered whether cells that belong to different classes, and which were co-recorded in the same FOV, are randomly spaced relative to each other (intermingled) or occupy separate territories within MEC. The anatomical arrangement of the various cell types in MEC often appeared dispersed across single FOVs (see example cell maps in Fig. 4*A*).

To investigate, we quantified the mutual anatomical distances between cell somata over all combinations of cell types at the single-session (i.e., single-FOV) level (i.e., pairwise comparisons between HD, grid, border, and OV cells; see example in *SI Appendix*, Fig. S5*A*) after masking out regions that were visibly not part of MEC based on tdTomato expression patterns (*SI Appendix*, *Extended Methods*). We defined starter cell populations (functional cell types above cutoff) for each cell class in each session and quantified nearest-neighbor (NN) distances between size-matched groups (containing the same number of neurons) from each class (Fig. 4*B*; two starter populations “A” and “B”; minimum number of cells in each cell class: 5).

We observed that within MEC the somata of grid and HD, grid and OV, and grid and border cells often segregated anatomically from each other (Fig. 4 *C*, *Top* cell maps), while HD and OV, HD and border, and OV and border cells seemed to intermingle (Fig. 4 *C*, *Bottom* cell maps). To quantify, we compared the distribution of interclass NN distances with a size-matched reference distribution, which we constructed by randomly permuting cell IDs for all cells in each session (“Shuffled”). Grid/HD, grid/OV, and grid/border distributions frequently appeared shifted toward larger NN distances compared with the reference distribution (cumulative distributions and boxplots in Fig. 4*C*) as would be the case if cell classes were anatomically more separated from each other than expected by chance. To compare across all recordings and animals, we normalized the average distances to the reference distribution in each recording (Fig. 4*D*). Values above 1 indicate that the average interclass NN distances (e.g., grid versus HD NN distances) are larger than those in the reference distribution. We found that the observed trends (examples shown in Fig. 4*C*) were maintained when compared across all recordings, in that grid/HD, grid/OV, and grid/border distributions were shifted above 1 (mean ± SD, grid/HD 1.13 ± 0.18, grid/OV 1.08 ± 0.12, grid/border 1.15 ± 0.24), indicating anatomical separation, while HD/OV, HD/border, and OV/border distributions were comparably closer to 1 (mean ± SD, HD/OV 1.02 ± 0.07, HD/border 1.04 ± 0.15, OV/border 1.01 ± 0.13), indicating anatomical intermingling of cell somata. Grid/HD distributions were statistically different from HD/OV, HD/border, and OV/border distributions, and grid/border distances were statistically different from those of HD/border (Fig. 4 *D*, *Left*). Grid/border, grid/OV, grid/HD, and HD/border distributions were significantly different from a population mean of 1 (Fig. 4 *D*, *Right*). We obtained similar results when analyzing the inter (between cell classes) to intra (within cell classes) NN distance ratios (*SI Appendix*, Fig. S5*B*) and filtering cells according to different spatial-directional score cutoffs (*SI Appendix*, Fig. S5*C*).

To summarize the results of the multiple pairwise intercell class comparisons, we constructed undirected graph networks from the data, which allowed us to visualize the pairwise distance relationships in a two-dimensional layout of nodes (Fig. 4*E*; same data as presented in Fig. 4*D*). Each node of these graph networks constitutes one cell class and the weight between each node is determined by the inverse of the normalized distance, such that cell class distances with larger normalized distances have lower weight and those with closer distances have higher weight. After random initialization of node positions, we then ran a simulation (“spring-loaded model”), which allowed the graph visualization to evolve at each iteration (1,000 iterations total) and either draw nodes closer together or push them farther apart depending on their weight relationships. Fig. 4 *E*, *Top* shows an example outcome of such a simulation, which shows grid cells separating from all other cell classes. Further examples are shown in Fig. 4 *E*, *Middle*. We defined a measure of separation as the ratio of the Euclidean distance between the grid node and center of the nodes of all other cell types (interdistance) over the mean distance between nodes of all other (nongrid) cell types (intradistance) (ratio). Over 1,000 shuffled results for which we randomly initialized weights before starting the simulation, the distance ratio distribution had a median close to 1 (0.8; Fig. 4 *E*, *Bottom*; see more examples of shuffled simulation outcomes in *SI Appendix*, Fig. S5*D*), while simulations run on the real data on average ended up with larger values (median 5.0; Fig. 4 *E*, *Bottom*). Increasing the number of iterations yielded even more extreme results (*SI Appendix*, Fig. S5*E*).

Taken together, we provide evidence for anatomical and functional separation of grid cells from all other known spatially modulated cell classes in superficial MEC.

### Grid Cells Cluster Anatomically.

**Fig. 5.**
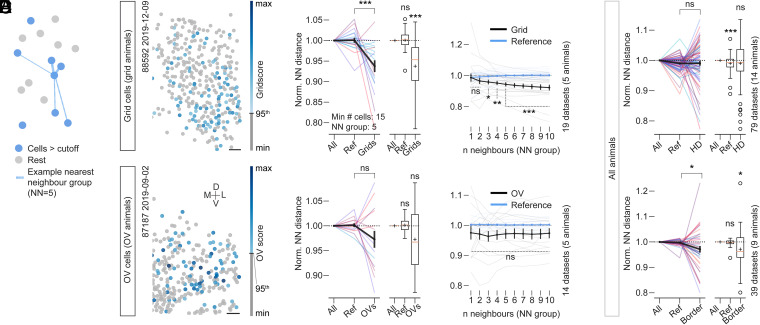
Grid cells cluster anatomically. (*A*) Schematic of NN analyses. The mean NN distances in *C* and *E* were extracted from groups of five starter cells (blue in schematic). Lines indicate distances to the five nearest neighbors of the example cell in the center. This group size (“NN group”) is systematically varied in *D*. (*B*–*D*) Grid animals (*Top*) and OV animals (*Bottom*). The minimum number of cells above the cutoff for each panel was set at 15. (*B*) Two sessions with multiple grid cells (“Grid animal”; *Top*) or multiple OV cells (“OV animal”; *Bottom*) (color-coded grid score or OV score); gray shows cells that did not meet cutoff criteria. (Scale bars, 50 µm.) (*C*) Normalized NN distances. (*C*, *Left*) Lines indicate recording sessions and colors represent animals (mean ± SEM is shown by a black line). (*C*, *Right*) Boxplots: Boxes extend from lower to upper quartiles; the orange line indicates the median, and whiskers indicate 1st to 99th percentile; outliers are shown as open black circles, and black plus signs indicate the mean. Statistics above line plots indicate results of two-sided Mann–Whitney *U* test, and above boxplots indicate two-sided Wilcoxon signed-rank test (against 1) (*P < 0.05, **P < 0.01, ***P < 0.001); ns, not significant (*P* > 0.05). See *SI Appendix*, *Extended Results* for detailed statistics. (*D*) Mean NN distance over varying numbers of neighbors (NN group) for grid cells in grid animals (*Top*) and OV cells in OV animals (*Bottom*). Thin black and blue lines show single recordings and thick lines show group average ± SEM. Significance (*P < 0.05, **P < 0.01, ***P < 0.001) indicates the result of two-sided Mann–Whitney *U* test, data vs. reference. Vertical labels show the total number of animals and sessions that were used in each comparison (for both *C* and *D*). See *SI Appendix*, *Extended Results* for detailed statistics. (*E*) Normalized, mean NN distances and statistics as in *C* for HD cells (*Top*) and border cells (*Bottom*) including all animals. See *SI Appendix*, *Extended Results* for detailed statistics.

We next wondered whether grid, border, HD, and OV cells each exhibit clustering over anatomical space like has previously been reported for grid cells in MEC (35, 36). To address this question, we compared NN distance statistics for groups of starter cells (functional cell types above the cutoff; minimum number of starter cells: 15) and reference cells (cells that were not part of the starter cell population, “Ref”) with statistics derived from cells that were picked randomly from all cells in each recording (“All”) (Fig. 5*A*; see example cell maps in Fig. 5*B*). We first masked out regions that were visibly not part of MEC (same procedure as for Fig. 4). Then, for each recording, we calculated the average NN distances over the five closest neighbors (NN group 5) of cells in all groups, which were size-matched to the starter cell population and normalized to All (normalized NN distances in Fig. 5 *C–E*). Values below 1 indicate distances that are smaller than those derived from randomly picked cells (All).

Similar to previous reports (35, 36), we observed that the NN statistics of grid cells indicated strong clustering in grid animals (Fig. 5 *C*, *Top*; grids mean ± SEM, 0.94 ± 0.01, same group of animals as in Fig. 3), which was maintained across cell inclusion thresholds (95th percentile shuffling cutoff in Fig. 5; 99th percentile shuffling cutoff in *SI Appendix*, Fig. S6 *A*, *Left*; grids mean ± SEM, 0.92 ± 0.02). When we varied the NN group sizes from 1 to 10 cells (Fig. 5 *D*, *Top*), clustering became statistically significant when groups were expanded beyond two neighbors and stayed robust over successively larger NN group sizes (Fig. 5*D*). This indicates that the observed clustering was not a consequence of doublets or triplets of cells but stable over large groups of neurons.

A similar trend was observed for OV cells in OV animals (same group of animals as in Fig. 3), which did, however, not reach statistical significance (see also *SI Appendix*, Fig. S6 *A*, *Right* for 99th percentile OV score cutoff, 0.97 ± 0.03) and was unstable over varying group sizes (Fig. 5 *C* and *D*, *Bottom*; OV mean ± SEM, 0.97 ± 0.02). Border and HD cells exhibited distance relationships that were less conclusive overall. While HD cells showed an overall dispersed arrangement (Fig. 5 *E*, *Top*; statistics over all animals; HD mean ± SEM, 0.99 ± 0.01; *SI Appendix*, Fig. S6*A*; 99th percentile cutoff, 1.00 ± 0.01), border cells did show some degree of clustering (Fig. 5 *E*, *Bottom*; statistics over all animals; border mean ± SEM, 0.97 ± 0.01), but it appeared less stable over varying cutoffs (*SI Appendix*, Fig. S6*A*; 99th percentile cutoff, 0.98 ± 0.02). Similar results were obtained when the NN distance analysis over groups of cells with a fixed number of neighbors was substituted with an analysis of all pairwise distances between starter cells (median pairwise distances over all cell pairs; *SI Appendix*, Fig. S6 *C–F*). For this analysis, we also varied the cutoff for the minimum number of starter cells (*SI Appendix*, Fig. S6*D*) and subsequently included all animals in the analysis (*SI Appendix*, Fig. S6*E*; no preselection for grid or OV animals), neither of which changed the results.

All in all, we were able to confirm previous reports of clustering in grid cells and extended this analysis over all other spatially modulated cell types. While some cell classes like OV and border cells did show trends for decreased NN distances compared with a reference population, none of the other cell classes exhibited anatomical clustering as strongly as grid cells.

### Grid, HD, and Border Cells Cover Stable Anatomical Territories along the MEC/PAS Border.

Upon closer investigation of our implantation sites and tdTomato labeling patterns in vivo, we noticed that animals in which we recorded an abundance of grid cells were often implanted quite medially, that is, close to the boundary between MEC and the more medially located PAS. In these animals (“MEC/PAS animals”), which we frequently recorded over weeks, clear and stable demarcations in the tdTomato channel were discernible, with cells in superficial MEC showing strong labeling in this channel compared with neighboring, more medial structures (see example in *SI Appendix*, Fig. S7*A*; see overview of all implant positions and tdTomato border visibility in *SI Appendix*, Fig. S2 *H*, *Right*). This matches descriptions of anatomical projection patterns from superficial MEC to ipsilateral hippocampus (51). Moreover, we noticed that the fractions of functional cell types stayed relatively stable across recording days, with grid, HD, and border cells showing roughly similar population ratios (*SI Appendix*, Fig. S7*B*), indicating that our recording locations and the recorded networks themselves remained stable.

**Fig. 6.**
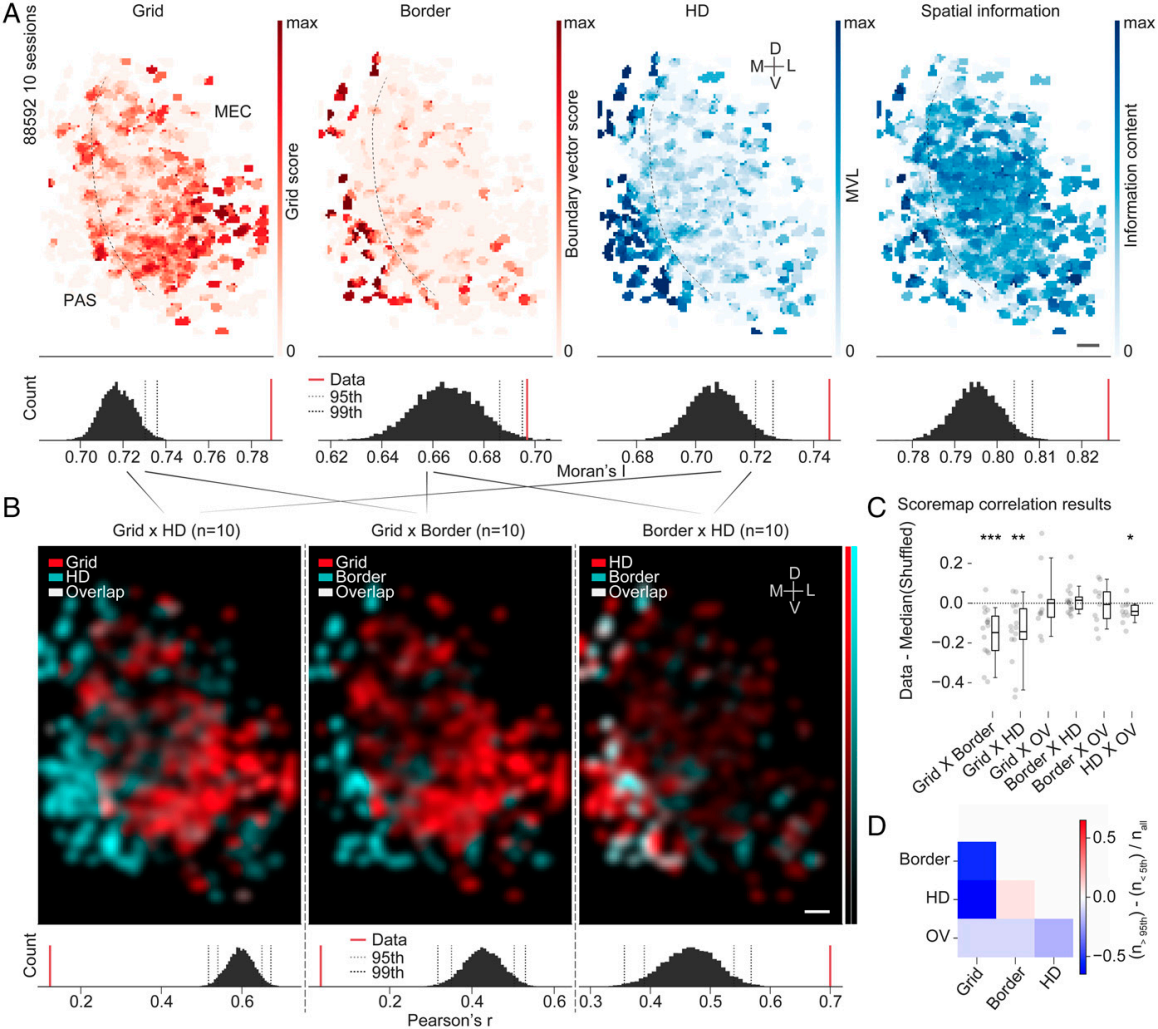
Functional cell types cover distinct and stable anatomical territories. (*A*) Four example topographic tuning maps, color-coded by score, are shown for one animal with multiple FOVs aligned as in *SI Appendix*, Fig. S7*C*. *Left* to *Right*: grid score (Grid), boundary vector score (Border), HD tuning expressed as MVL (HD), and spatial information content (“Spatial information”). (*A*, *Bottom*) Distribution of shuffled Moran’s I values for each tuning map and actual values (“Data”; red); 95th and 99th percentile cutoffs are shown as dashed lines. (Scale bar, 50 µm.) (*B*) Overlay and correlation of topographic tuning maps for different tuning properties. Maps were first smoothed with a Gaussian kernel with sigma 2 bins. The number of combined sessions in each example is shown above (*n*). Diagonal lines indicate which maps were combined and smoothed (composites in *A*). (*B*, *Bottom*) Pearson’s correlation (Pearson’s *r*) between the different tuning maps, compared with a shuffled distribution. Actual values are shown in red (Data), and 95th and 99th percentile cutoffs are shown as dashed lines. (Scale bar, 50 µm.) (*C*) Topographic tuning map correlation results (dots indicate animals). Data are shown as the difference of the actual Pearson’s *r* value (Data) and the median of the shuffled distribution of Pearson’s *r* values (“Shuffled”). Statistics at the top of the figure indicate statistically significant (*P* < 0.05) results of two-sided Wilcoxon signed-rank test against a mean of zero (*P < 0.05, **P < 0.01, ***P < 0.001). See *SI Appendix*, *Extended Results* for detailed statistics. (*D*) Summary of topographic tuning map correlation results. Data are shown as a color-coded representation of the difference in the number of results that are above the 95th percentile shuffling cutoff and the number of results that are below the 5th percentile cutoff over all sessions (same data as analyzed in *C*). Grid × border −0.53, grid × HD −0.67, grid × OV −0.1, border × HD 0.07, border × OV −0.1, HD × OV −0.2. Grid/border as well as grid/HD territories are most strikingly anticorrelated compared with the rest.

We thus wondered if and how the observed anatomical patterns overlapped with the distribution of functional cell types in these regions. To investigate this question, we first aligned the FOVs of as many recordings as possible for each animal with each other based on anatomical landmarks (mean number of FOVs per animal: 8.6; *n* = 15 animals) (*SI Appendix*, Fig. S7*C*). This way, we were able to create composites with high anatomical congruence (*SI Appendix*, Fig. S7 *C*, *Middle*; structural similarity index measure, SSIM), aligned across multiple recordings that were spread over many days in each animal (see example in *SI Appendix*, Fig. S7 *C*, *Bottom*). We manually annotated MEC and neighboring structures in every FOV (see overview in *SI Appendix*, Fig. S7 *D*, *Left*) and created topographic tuning maps from all neurons by first binning each cell’s tuning strength (e.g., its grid score, etc.) in anatomical space, and then projecting those binned maps over all aligned sessions (difference between first and last session, mean ± SD, 22.1 ± 12.9 d, *n* = 15 animals). Examples can be seen in Fig. 6*A* for one MEC/PAS animal (10 aligned sessions; the boundary is indicated by a stippled gray line in each topographic tuning map). In animals in which this clean anatomical boundary was discernible, grid cells cluster on the MEC side (more examples are shown in *SI Appendix*, Fig. S7*D*). Sharp transitions occurred toward neighboring PAS in which border and HD cells dominated (Fig. 6*A*). To assess whether these functional transitions were more abrupt than expected by chance and hence whether stable anatomical territories were formed by either cell type, we calculated a global autocorrelation statistic (Moran’s I; Fig. 6*A* and *SI Appendix*, Fig. S7*D*), which we compared with shuffled distributions of the same data. The shuffled distributions were constructed from projections of the same underlying cell position data after scrambling the tuning and region of interest (ROI) associations. These shuffled topographic tuning maps thereby mimicked a “salt and pepper” organization across the tissue. The analysis showed that the anatomical expression patterns of functional cell types across MEC and PAS were more structured than expected by chance (Fig. 6 *A*, *Bottom*; see *SI Appendix*, Fig. S7*D* for five animals that were implanted at a similar location). In addition, grid modules appeared clustered according to gradients described in the literature (38, 40, 41), with smaller-spaced grid cells located more dorsally than larger-spaced ones (see *SI Appendix*, Fig. S8 for an example).

Furthermore, the territories of grid, border, and HD cells appeared spatially anticorrelated at the MEC/PAS boundary (Fig. 6 *B*, *Bottom* shows Pearson’s correlation between smoothed topographic tuning maps compared with shuffled distribution). We compared the strength of this effect across all animals, including those that were not implanted close to the PAS boundary, by subtracting the median of all shuffled Pearson’s correlation values from the data (Fig. 6*C*; values below zero indicate that expression patterns were more anticorrelated than expected by chance). To summarize these data, we compared the number of results above the 95th percentile shuffling distribution cutoff (i.e., topographic tuning maps that were most strongly correlated) with those that were below the 5th percentile shuffling distribution cutoff (i.e., those that were most strongly anticorrelated across all cell classes) (discrimination ratio; Fig. 6*D*). In line with our observations of functional territories in MEC/PAS animals and our analyses of cell soma distribution patterns on the single-session level (Fig. 4), grid cell territories were most stably distinct from HD and border cell territories, while OV, border, and HD territories appeared to overlap more (Fig. 6*C*; mean ± SD, grid × border −0.15 ± 0.13 [15 animals]), grid × HD −0.14 ± 0.15 [15 animals]), grid × OV 0.00 ± 0.16 [10 animals]), border × HD 0.01 ± 0.07 [15 animals]), border × OV −0.01 ± 0.10 [10 animals]), HD × OV −0.04 ± 0.05 [10 animals]). Because of the low coexpression level of grid and OV cells in the FOVs that were accessible to us in this study, the analysis of anatomical territories was less powerful in the grid × OV case (Fig. 6 *C* and *D*), although our previous analyses predict strong anticorrelation of grid and OV distribution patterns at larger anatomical scales. Future studies that make use of further improvements, enabling both the enlargement of single-session FOVs as well as the systematic shifting of FOVs between sessions, may extend the present investigations to even larger anatomical spaces (46).

The robustness of the observed effects indicates that functional cell types occupy anatomically stable territories over time, with the allocation of grid and HD/border networks on either side of the MEC/PAS boundary leading to the most apparent and stable topographic distribution differences.

## Discussion

We have shown that with a new generation of 2P miniscopes (46), we can systematically investigate the topographic arrangement of all known spatially modulated cell types in the superficial layers of MEC and adjacent PAS. The lightweight and flexible connection cable of the new miniscope is on par with that of previous 1P miniscopes and its spatial resolution approaches that of 2P benchtop setups. Our data demonstrate that 2P miniscopes enable the simultaneous recording of well over a hundred cleanly isolated cells at high SNRs while mice engage in unrestrained, exploratory behavior in large open-field arenas over tens of minutes. Future studies using upgraded 2P miniscopes (46) will benefit from further increases in the total number of recordable cells per FOV as well as the ability to systematically expand surveys over multiple layers and multiple neighboring anatomical regions in the same animal.

We report that grid and OV cells in MEC display low co-occurrence on the anatomical scales that were accessible to us in this study. Animals segregate into groups with either high numbers of grid cells or high numbers of OV cells per FOV. This is unlikely to be a result of sampling different layers within MEC, since our recordings were mostly obtained from layer II or at the border between layers II and III and, even if they varied slightly from day to day, the observed anticorrelations on the population level between OV and grid cells remained stable across recordings and over days. Moreover, we did not find any correlation between the fraction of OV or grid cells and numbers of tdTomato-positive cells, the latter of which were more numerous in layer III than layer II in our study. Together, this suggests that grid and OV cells occupy largely separate territories in the parahippocampal region and might indicate that grid and OV cells are supported by networks with distinct principal and interneuron connectivity motifs. Such differences in connectivity have previously been proposed for networks supporting the emergence of aperiodic spatial cells and grid cells in MEC (52), which might be relevant if OV cells are at least to some extent contained within the miscellaneous class of aperiodic spatial cells (53). The segregation of OV and grid cells might thereby reflect the different affordances of underlying network computations. These involve, in the case of grid cells, the formation of internally generated spatial firing patterns anchored to the overall layout of the environment the animal is in (20, 54) and, in the case of OV cells, the formation of firing fields that anchor to and move flexibly with objects within that same environment (24). Although we were not able to resolve the exact number and location of grid or OV territories in this study, future studies using technological advances, such as 2P miniscopes with even larger FOVs (46), will extend the anatomically observable areas and thereby help to elucidate these large-scale organizational principles further.

The somata of grid cells cluster within MEC, which is in line with previous reports (35, 36). This topographic pattern seems to go along with the anatomical separation from various other cell classes, visible within MEC and in between MEC and neighboring PAS. Although direct principal-to-principal neuron connectivity is overall low in the superficial layers of MEC (28, 55, 56), the dense anatomical packaging of grid cells may lead to elevated functional connectivity between them (11), which is to be expected if grid cells are organized in CANs (27–29, 31). Here, we extend these measurements to all other spatially modulated cell classes and observe that, while trends for anatomical clustering do exist in some cell classes, namely OV and border cells, only grid cells seem to stably aggregate in groups of three or more cells. While we cannot rule out that, for example, OV and border cells might display more apparent clustering in different anatomical regions and over larger samples, this result could indicate that HD, border, and OV cells form more loosely organized attractors or might instead partially inherit their intrinsic dynamics from upstream networks, which would in turn alleviate the need for close anatomical packing within MEC itself. It may, however, also indicate that these cell types are embedded within the same densely interconnected network. Indeed, we observe that OV, border, and HD cells appear anatomically intermingled, resembling a salt and pepper organization. This more homogeneous distribution, and thereby the anatomical closeness across compared to within types, might favor strong communication between cell types (11), which may be more difficult to maintain if cells were arranged in anatomically isolated clusters instead. This might be necessary for the emergence and maintenance of firing patterns in these three cell types. In line with this argument, recent studies (57–60) report that the combination of allocentric HD coding and egocentric information in relation to environmental boundaries might have an important role in forming border and boundary vector cell firing fields. Border and HD cells might thus benefit from anatomical closeness and thus potentially dense information exchange between each other. Similar mechanisms might require OV cells to maintain anatomical proximity to cells tuned to HD and environmental boundaries. Future studies that combine 2P miniscope imaging with single-cell optogenetic stimulation protocols in functionally identified neuronal subtypes might be able to elucidate these connectivity motifs and enable detailed comparisons with results obtained in HD networks in fruit flies, where HD tuning can be monitored across entire ensembles of cells with known connectivity (14–16). This will help to elucidate principles underlying the functional and anatomical organization of space circuits across different taxa.

Our data indicate that grid neural networks do form largely isolated entities in the superficial layers of MEC, which may isolate them from direct connectivity with other spatially modulated cell classes. In line with this, we found reliable “hotspots” of grid cells in MEC at the border with PAS across animals, which appeared to span large parts of the imaged FOVs. PAS seemed to be populated with mostly HD and border cells and fewer, more scattered grid cells, which is in line with previous reports (61, 62). Although grid cells in MEC are in close contact with HD and border cells in superficial PAS, they respect a strikingly clean anatomical boundary that is visible across animals and, within animals, remains stable over multiple days and weeks. While the sampling of slightly varying cell populations across days in our study precluded the systematic investigations of single-cell tuning stability, future studies using 2P miniscope recordings in MEC and PAS will be able to clarify whether single cells maintain stable tuning properties over time and, if not, whether drift occurs on the same timescale as has been observed in functional imaging studies of place cells in hippocampus (63–65). Together with the observation of anatomical segregation within MEC, our observations hint at a relatively rigid, developmentally controlled process, which leads to the formation of highly structured intrinsic networks in the parahippocampal region. The anatomical segregation of clustered grid networks on the one hand and intermingled border and HD networks on the other hand might be seeded during development of the parahippocampal network, in line with observations that the maturation of grid cells is protracted compared with both HD and border cells (66–69). HD, border, and potentially OV cells, for which the developmental time course has not yet been explored, might form early, intermingled subnetworks that foster the establishment of grid networks. Future studies employing 2P miniscope imaging in combination with clever labeling of genetically accessible subpopulations might yield insights into how this internal organization is orchestrated during development, and specifically how functional arrangements follow known anatomical developmental patterns in this region (70, 71).

## Materials and Methods

Adult male mice were implanted with a customized 1-mm-diameter GRIN lens attached to a 1-mm-square prism, or with 1.3 × 1.3 × 1.6 mm prisms alone. For virus injection and implantation procedures, see *SI Appendix*, *Extended Methods*. Custom, lightweight 2P miniscopes were fabricated from machinable plastic and connected to 1) the laser, 2) the detection module containing photomultiplier tubes, and 3) the MEMS scanner controller via a lightweight and flexible connection cable (for details, see *SI Appendix*, *Extended Methods*). Imaging sessions were initiated by briefly head fixing the animal on a treadmill, which enabled safe fixation of the miniscope and finding suitable FOVs to record from. Positions and head angles of the animal inside square open-field arenas were tracked via two colored light-emitting diodes attached to the miniscope, in sync with the imaging. A detailed description of the imaging setup, the 2P miniscope, behavioral recording, and synchronization routines can be found in *SI Appendix*, *Extended Methods*. Data were analyzed using a custom-written analysis pipeline based on the DataJoint framework (72). For details of the analysis methods, including description of spatial scores, NN analyses, and shuffling procedures, see *SI Appendix*, *Extended Methods*.

All experiments were performed in accordance with the Norwegian Animal Welfare Act and the European Convention for the Protection of Vertebrate Animals Used for Experimental and Other Scientific Purposes (Permit Nos. 18013, 6021, and 7163).

## Supplementary Material

Supplementary File

## Data Availability

Data for this paper was deposited in NIRD Research Data Archive (DOI: 10.11582/2022.00005). The code for analyses and figures is provided in Zenodo (DOI: 10.5281/zenodo.5910807).
