# Supplementary material for: Functional network topography of the medial entorhinal cortex
Source: Proc Natl Acad Sci U S A. 2022 Feb 8;119(7):e2121655119. doi: 10.1073/pnas.2121655119 (PMC8851479; doi:10.1073/pnas.2121655119)
Supplement: Supplementary File [file pnas.2121655119.sapp.pdf]

Supplementary Information for
Functional network topography of the medial entorhinal cortex

Horst A. Obenhaus[1]*, Weijian Zong[1], R. Irene Jacobsen[1], Tobias Rose[2], Flavio Donato[3], Liangyi Chen[4], Heping Cheng[4], Tobias Bonhoeffer[5], May-Britt Moser[1]*, Edvard I. Moser[1]*

* Horst. A. Obenhaus, Edvard I. Moser
Email: horst.obenhaus@ntnu.no, edvard.moser@ntnu.no

This PDF file includes:

Extended Results
Extended Methods
Figures and figure legends S1 to S8

Extended Results

Statistics pertaining to analyses in Fig. 3 D

In Fig. 3D we correlate the fractions of each cell type across all sessions using the Spearman correlation coefficient (e.g., fraction of grid cells vs. fraction of OV cells). Each pair of comparisons is color coded by Spearman's r and the r value is shown as text in the center of the box. Results are filtered by anatomical location (MEC only), using a minimum cutoff of 15 cells. Only "pure" cells (i.e., those cells that cross cell selection criteria for only one cell type) were considered. Correlations between fractions of cell types were as follows: Grid vs. Border: r=-0.35, $n_{Grid}=n_{Border}=124$, p=5.47e-05***; Grid vs. HD: r=-0.38, $n_{Grid}=n_{HD}=124$, p=1.27e-05***; Grid vs. OV: r=-0.596, $n_{Grid}=n_{OV}=36$, p=0.00013***; OV vs. Border: r=0.34, $n_{OV}=n_{Border}=36$, p=0.046*; OV vs. HD: r=0.23, $n_{OV}=n_{HD}=36$, p=0.17 ns; HD vs. Border: r=0.16, $n_{HD}=n_{Border}=124$, p=0.084 ns.

Statistics pertaining to analyses in Fig. 3 F

To assess whether the differences in cell fractions and discrimination indices were bigger than expected by chance, we created a shuffled distribution as described in the Results (Section "*Object vector cells and grid cells segregate* "). In creating the shuffled distribution, we labeled a subset of all cells as either grid or OV cells, matching the observed combined frequency of grid and OV cells in our data and chose a ratio of 50% grid and 50% OV cells, mimicking a balanced population ratio. We then drew random subsets from the total cell population that matched the average population size of our recorded sessions, which were then classified as either "OV" or "Grid", depending on which cell type was represented more often. Fig. 3F right shows the fraction of cell types (OV or Grid) and discrimination indices in the data in comparison to a shuffled distribution. Stars ('*') indicate results of the two-sided Wilcoxon signed-rank tests of the data versus the population mean of the shuffled data (* p<0.05, ** p<0.01, *** p<0.001). Grid animals (top): fraction grid, Z=28, $n_{Grid}=18$, p=0.010*; fraction OV Z=1, $n_{OV}=18$, p=1.53e-05***; discrimination index Z=0, $n_{Disc.Index}=18$, p=7.63e-06***. OV animals (bottom): fraction grid, Z=3, $n_{Grid}=16$, p=0.00015***; fraction OV Z=23, $n_{OV}=16$, p=0.018*; discrimination index Z=0, $n_{Disc.Index}=16$, p=3.05e-05***.

Statistics pertaining to analyses in Fig. 4 D

Comparison of mean nearest neighbor (NN) distances across cell types. To compare across all recordings and animals we normalized the average distances to the reference distribution in each recording. Left: Kruskal-Wallis H-test across all groups: H=14.504, n=6 groups, p=0.013*; statistics on top of figure indicate results of Mann Whitney U test. The test statistic was obtained from results of individual, pairwise comparisons; 95th percentile shuffling cutoffs were used throughout). Group differences in NN distances were as follows: Grid/HD vs. Grid/OV, U=372, $n_{Grid/HD}=56$, $n_{Grid/OV}=12$, p=0.57 ns; Grid/HD vs. Grid/Border, U=1457, $n_{Grid/HD}=56$, $n_{Grid/Border}=51$, p=0.86 ns; Grid/HD vs. HD/OV, U=627, $n_{Grid/HD}=56$, $n_{HD/OV}=16$, p=0.016*; Grid/HD vs. HD/Border, U=2472, $n_{Grid/HD}=56$, $n_{HD/Border}=68$, p=0.0044**; Grid/HD vs. OV/Border, U=612, $n_{Grid/HD}=56$, $n_{OV/Border}=16$, p=0.027*; Grid/OV vs. Grid/Border, U=282, $n_{Grid/OV}=12$, $n_{Grid/Border}=51$, p=0.68 ns; Grid/OV vs. HD/OV, U=131, $n_{Grid/OV}=12$, $n_{HD/OV}=16$, p=0.11 ns; Grid/OV vs. HD/Border, U=497, $n_{Grid/OV}=12$, $n_{HD/Border}=68$, p=0.23 ns; Grid/OV vs. OV/Border, U=128, $n_{Grid/OV}=12$, $n_{OV/Border}=16$, p=0.14 ns; Grid/Border vs. HD/OV, U=533, $n_{Grid/Border}=51$, $n_{HD/OV}=16$, p=0.067 ns; Grid/Border vs. HD/Border, U=2151, $n_{Grid/Border}=51$, $n_{HD/Border}=68$, p=0.025*; Grid/Border vs. OV/Border, U=539, $n_{Grid/Border}=51$, $n_{OV/Border}=16$, p=0.055 ns; HD/OV vs. HD/Border, U=522, $n_{HD/OV}=16$, $n_{HD/Border}=68$, p=0.81 ns; HD/OV vs. OV/Border, U=135, $n_{HD/OV}=n_{OV/Border}=16$, p=0.81 ns; HD/Border vs. OV/Border, U=588, $n_{HD/Border}=68$, $n_{OV/Border}=16$, p=0.62 ns; Right: Color coded average of results on the left. Stars indicate results of two-sided one sample t-test against population mean of 1 (p<0.05 *, p<0.01 **, p<0.001 ***); Grid/HD: 12 animals, t=5.364, $n_{Grid/HD}=56$, p=1.67e-06***; Grid/OV: 5 animals, t=2.211, $n_{Grid/OV}=12$, p=0.049*; Grid/Border: 12 animals, t=4.324, $n_{Grid/Border}=51$, p=7.31e-05***; HD/Border: 13 animals, t=2.260, $n_{HD/Border}=68$, p=0.027*; HD/OV: 6 animals, t=1.065, $n_{HD/OV}=16$, p=0.30 ns; OV/Border: 7 animals, t=0.395, $n_{OV/Border}=16$, p=0.7 ns;

Statistics pertaining to analyses in Fig. 5 C

NN distance statistics were obtained for groups of starter cells (functional cell types above cutoff, minimum number of starter cells: 15) and reference cells (cells that were not part of the starter cell

population, “Ref”). This was compared to statistics derived from cells that were picked randomly from all cells in each recording (“All”). Groups were size matched to the starter cell population and normalized to distributions of randomly picked cells (“All”). Statistics on top of line plots indicate results of two-sided Mann-Whitney U test, and on top of box plots indicate two-sided Wilcoxon signed-rank test (against 1.) (p<0.05 *, p<0.01 **, p<0.001 ***); ns not significant (p>0.05). Grid: data over 5 animals, Mann-Whitney U=308, $n_{Ref}=n_{Grid}=19$, p=0.00021*** two-sided; Wilcoxon signed-rank test (against 1.): Ref: Z=90, $n_{Ref}=19$, p=0.86 ns, Grid: Z=9, $n_{Grid}=19$, p=0.00013***. OV: data over 5 animals, Mann-Whitney U=131, $n_{Ref}=n_{OV}=14$, p=0.14 ns two-sided; Wilcoxon signed-rank test Z=30, $n_{OV}=14$, p=0.17 ns two-sided.

Statistics pertaining to analyses in Fig. 5 D

For this figure we systematically varied the number of NN (NN group) from 1 to 10 and pursued NN analyses as described for Fig. 5C. Significance (p<0.05*, p<0.01**, p<0.001***) indicates the result of the two-sided Mann-Whitney U test Data vs. Reference. Mann-Whitney U-test results: Grid vs. Ref, $n_{Ref}=n_{Grid}=19$; NN=1 U=147, p=0.34 ns; NN=2 U=115, p=0.058 ns; NN=3 U=103, p=0.025*; NN=4 U=89, p=0.0079**; NN=5 U=66, p=0.00087***; NN=6 U=53, p=0.00021***; NN=7 U=49, p=0.00013***; NN=8 U=50, p=0.00015***; NN=9 U=48, p=0.00012***; NN=10 U=46, p=9.15e-05***; OV vs. Ref: $n_{Ref}=n_{OV}=14$; NN=1 U=77, p=0.35 ns; NN=2 U=66, p=0.15 ns; NN=3 U=65, p=0.14 ns; NN=4 U=69, p=0.19 ns; NN=5 U=72, p=0.24 ns; NN=6 U=65, p=0.14 ns; NN=7 U=67, p=0.16 ns; NN=8 U=64, p=0.12 ns; NN=9 U=66, p=0.15 ns; NN=10 U=68, p=0.18 ns; ns: not significant (p>0.05).

Statistics pertaining to analyses in Fig. 5 E

Normalized, mean nearest neighbor (NN) distances as in Fig. 5C for head direction (HD) cells (top, 95th percentile shuffling cutoff) and border cells (bottom, 95th percentile shuffling cutoff) including all animals. HD: data over 14 animals, Mann-Whitney U=2887, $n_{Ref}=n_{HD}=79$, p=0.42 ns two-sided; Wilcoxon signed-rank test (against 1.): Ref: Z=894, $n_{Ref}=79$, p=0.0008*** two-sided, HD: Z=1467, $n_{HD}=79$, p=0.58 ns, two-sided. Border: data over 9 animals, Mann-Whitney U=1005, $n_{Ref}=n_{Border}=39$, p=0.015* two-sided; Wilcoxon signed-rank test (against 1.), Ref: Z=305, $n_{Ref}=39$, p=0.24 ns two-sided, Border: Z=222, $n_{Border}=39$, p=0.019*, two-sided; ns: not significant (p>0.05).

Statistics pertaining to analyses in Fig. 6 C

Pearson’s correlation between smoothed topographic tuning maps (Fig. 6B, bottom row) showed that the territories of grid, border, and HD cells appeared spatially anticorrelated at the MEC/PAS boundary. We compared the strength of this effect across all animals by subtracting the median of all shuffled Pearson’s correlation values from the data. Data are shown as the difference of the actual Pearson’s r value (“Data”) and the median of the shuffled distribution of Pearson’s r values (“Shuffled”). Kruskal Wallis H-test over n=6 groups p=0.0012**, H=20.18. Statistics on top of figure indicate statistically significant (p<0.05) results of two-sided Wilcoxon signed-rank test against mean of zero: GridxBorder: Z=4, $n_{GridxBorder}=15$, p=0.00043***; GridxHD: Z=9., $n_{GridxHD}=15$, p=0.002**; GridxOV: Z=21, $n_{GridxOV}=10$, p=0.56 ns; BorderxHD: Z=55, $n_{BorderxHD}=15$, p=0.80 ns; BorderxOV: Z=25, $n_{BorderxOV}=10$, p=0.85 ns; HDxOV: Z=8, $n_{HDxOV}=10$, p=0.049*.

Extended Methods

All experiments were performed at the Kavli Institute for Systems Neuroscience, Norwegian University of Science and Technology (NTNU).

Animals

All experiments were performed in accordance with the Norwegian Animal Welfare Act and the European Convention for the Protection of Vertebrate Animals used for Experimental and Other Scientific Purposes, permit numbers 18013, 6021 and 7163. Only adult male mice (age >3 months)

were used, which were either C57BL/6JBomTac mice ("wild-type", Cat#B6JBOM; RRID: IMSR_TAC:b6jbom, Taconic) or transgenic mice expressing GCaMP6s ubiquitously in excitatory cells under control of the CaMKII promoter (Camk2a-tTA; tetO-G6s) (1).
Wild-type and GCaMP transgenic mice were housed in social groups of 2-6 individuals per cage under a 12h light/12h darkness schedule, in a temperature- and humidity-controlled vivarium. Food and water were provided ad libitum. The animals were housed under conditions free from specific pathogens according to the recommendations set by the Federation of European Laboratory Animal Science Associations (FELASA) (2). For most in vivo imaging experiments, GCaMP transgenic mice were used. Due to observed physiological abnormalities that we observed in old (age >11 months) transgenic animals, only results from transgenic animals that were younger than 10.5 months at the time of recording are reported in this study (age at recording mean ± SD 29.57 ± 5.82 weeks, n=103 sessions).

Viruses

In a subset of animals (3 out of 15), a virus expressing GCaMP6m (AAV1-syn-GCaMP6m, titer: 3.43e13 GC/ml, Cat#AV-1-PV2823, UPenn Vector Core, University of Pennsylvania, USA) was injected into wild-type animals. Most animals (transgenics and wild-types, 13 out of 15 animals) were injected with retrograde AAV (3) carrying tdTomato into the hippocampus (AAVretro-CAG-tdTomato, #59462-AAVrg, Addgene).

Surgeries

For all surgeries anesthesia was induced by placing the subjects in a plexiglass chamber filled with isoflurane vapor (5% isoflurane, IsoFlo, Zoetis, in medical air, flow of 1 liters/minute). Surgery was performed on a heated surgery table (38°C) and air flow was kept at 1 liters/minute with 1.5–3% isoflurane as necessary based on depth of anesthesia, as determined by physiological monitoring of vital signs (breathing and heartbeat). After each procedure, subjects were allowed to recover in a heated chamber (33 °C) until they regained complete mobility and alertness.

Virus injection into the hippocampus

On the day of surgery, adult mice (P60-P120) were anesthetized, and analgesics were provided (Rymadil, Pfizer, intraperitoneal injection, 5 mg/kg or Metacam, Boehringer Ingelheim, 5 mg/kg; Temgesic, Indivior 0.05-0.1 mg/kg subcutaneous injection, 0.05 mg/kg; Marcain, Aspen, local subcutaneous injection, 1-3 mg/kg). Eyes were protected from drying out by applying eye ointment (Simplex, Actavis). Most animals (13 out of 15) received an injection of AAVretro-CAG-tdTomato into the hippocampus to retrogradely label projections from ipsilateral, superficial layers of MEC. For this, two drill holes (drill bit: Cat#1RF HP 330 104 001 001 005 from Hagen and Meisinger, Germany) were made over the left hemisphere at [mm, measured from bregma] AP 2.1, ML 1, targeting dentate gyrus (DG), and AP 2.1 mm, ML 2.1, targeting CA3; for animals 97045 and 97046 AP was changed to 1.7. After drilling and exposing the brain surface, the drill holes were immediately covered with drops of saline (NaCl 9 mg/ml, B. Braun Medical). The virus was injected at the same coordinates as the drilled holes, at DV 1.8 and DV 1.7 for targeting the DG and CA3, respectively, at a rate of about 50-70 nl per minute via pulled glass micropipettes (Cat#504949, World precision instruments). The total injection volume ranged from 150 to 300 nl per injection site. After each injection was completed, the glass pipette was left in place for 10 minutes to give the virus time to diffuse before retraction. The drill holes were covered with UV curable cement (Venus Diamond Flow, Kulzer). Virus injections into the hippocampus were typically combined with MEC virus injections and implantations (see below). In rare cases where these procedures were split over weeks, the skin was sutured (Supramid DS 13, Resorba Medical, Germany), and the animal allowed to recover in a heated chamber (33°C, 30-90 minutes).

Virus injection into MEC and GRIN / prism implantation

In most cases the injections described above (AAVretro-CAG-tdTomato, hippocampus) were combined with virus injections and chronic implantation of GRIN / prisms into MEC. To gain access to MEC a large circular craniotomy (diameter ~ 4 mm) was made over the left hemisphere. For this, the skull was carefully leveled using bregma and lambda as measurement points and two reference drill points were created at [mm] 3.3 and 3.8 ML on the lambdoid suture. Then a craniotomy was

drilled around the reference points, covering mostly the left interparietal bone, with about one fourth of it stretching over to the left parietal bone. The bone was carefully lifted, exposing the brain surface and transverse sinus. The implantation side was frequently irrigated with saline (NaCl 9 mg/ml, B. Braun Medical) and small bleedings were stopped with highly absorbent sponges (Sugi Eyespear, Cat#30601, Questalpha, Germany). For virus injections into MEC, a Hamilton syringe (World Precision Instruments, microliter syringe #75, Hamilton Company) was used. Two injection sites were chosen at [mm] 3.2-3.3 ML and 3.8 ML with the frontal edge of the transverse sinus acting as reference for these coordinates: The medial injection site was offset about .25 mm (towards anterior) and the lateral about .2 mm and the syringe was angled at 9-10 degrees (tip towards posterior). Small incisions were then made in the dura with a syringe needle just underneath where the syringe tip interfaced with the brain surface and the injection needle was inserted and slowly progressed until it touched dura or bone. The needle was then retracted about 0.1-0.2 mm to target superficial layers of MEC and 400-500 nl virus was injected at a rate of 80 nl/min across two depths, spaced about 150-250 µm apart. After each injection the needle was left in place for 10 minutes before retraction.

The craniotomy was then protected by applying Kwik-Cast (World Precision Instruments) and the rest of the exposed skull was covered with a bonding agent (OptiBond All-In-One, Kerr). After removal of Kwik-Cast, the dura was sliced along the transverse sinus and a custom implant holder, attached to a stereotactic micromanipulator (1760, Kopf, CA, USA), clamping a customized 1 mm diameter GRIN (gradient-index) lens attached to a 1 mm square prism (total length approximately 4.7 mm, optimized for 920 nm, Grintech, Germany), was slowly lowered at an angle of 10-15 degrees (tip towards anterior) such that the prism entered the space between MEC and cerebellum. No aspiration of cerebellar tissue was performed. Once the prism was lowered such that it was visibly below the superficial cortex, it was then pressed forward about 0.5 mm to ensure optimal adherence of its frontal facing surface to the surface of MEC. Similar procedures were used for the implantation of a glass prism (1.3x1.3x1.6 mm, Sunlight, Fuzhou, China), which was glued to a 5 mm diameter cover glass (CS-5R, Warner, MA, USA) and held in place via a custom-made titanium cannula (diameter: 5 mm, 1 mm wall height). Cannula, cover glass and prism were glued together using UV curable adhesive (NOA61, Norland, NJ, USA). The exposed brain around the implantation site was covered with Kwik-Sil (World Precision Instruments) and the clamped implants were carefully fixed to the surrounding bone with UV curable cement (Venus Diamond Flow, Kulzer). A custom-designed titanium headbar was attached and centered on the dorsal surface of the skull and aligned parallel to the top face of the GRIN lens. All exposed areas of the skull, including the headbar, were then covered with dental cement (Paladur, Kulzer), made opaque by adding 0.5 g of carbon powder (Sigma Aldrich, CAS number 7440-44-0). After the surgery, the animals were allowed to recover in a heated chamber (33°C, 30-90 minutes) until they regained complete mobility and alertness.

Histology
Mice received an overdose of sodium pentobarbital (Apotekforeningen, 100mg/ml) before transcardial perfusion with freshly prepared paraformaldehyde (PFA, CAS Number: 30525-89-4, Alfa Aesar, 4% in phosphate-buffered saline (PBS), P3813, Sigma-Aldrich, Germany). After perfusion, the brain was extracted from the skull and kept in PFA 4% and at 4 degrees Celsius for post fixation. Samples were then sliced on a cryostat (CryoStar NX70, Thermo Scientific, USA, 30-50 µm thick sagittal sections) and either mounted directly on custom made gelatin coated glass slides (for cresyl violet (Nissl) stainings) or collected sequentially in a 24 well plate in PBS (for immunofluorescence). To determine implant positions and to identify which brain regions were covered by the prisms (see summary across all animals in SI Appendix, Fig. S2H), histology was aligned to the Allen Mouse Brain Common Coordinate Framework (CCFv3) (4) via an open-source python based tool (brainreg-segment) that allows for manual annotation of implant positions in a common coordinate space (5) and analysis of brain regions that are covered by the implants.

Immunofluorescence stainings
Brain slices (30-50 µm thick sagittal sections) were incubated for 1 h at room temperature (RT) in blocking buffer (2% gelatine, 2% BSA (bovine serum albumin, Cat#a2153, CAS Number: 9048-46-

8, Sigma), 0.1% Triton X-100 (Merck) in PBS). Primary antibodies diluted in blocking solution were added for an overnight incubation at room temperature (RT). After washing with a 1:3 dilution of the blocking buffer in PBS, slices were transferred to a fresh blocking buffer containing fluorophore-conjugated secondary antibodies for 1.5 hours at RT. After a final wash with PBS, slices were mounted in their appropriate anatomical order on glass slides (Polysine adhesion slides (Cat#10219280, Brand: J2800AMNZ, Thermo Scientific, US) using Prolong antifade with DAPI (Cat#P36935, Invitrogen). Primary antibodies used: mouse anti-NeuN, MAB377 (Merck), rabbit anti-GFP, A-11122 (Thermo Fisher). Secondary antibodies used: Alexa Fluor 488, donkey anti-rabbit IgG, A-21206 (Thermo Fisher); Alexa Fluor 647 rabbit anti-mouse IgG, ab169348 (abcam). For confocal imaging, a Zeiss LSM 880 microscope (Carl Zeiss, Germany) was used. Images were then acquired as z-stacks using an EC Plan-Neofluar 20×/NA 0.8 air immersion, 40x/NA 1.4 oil immersion (Cat#420762-9900, Zeiss Plan-Achromat, Zeiss, Germany) (Zeiss, laser power: 2-15%).

2-photon imaging setup

Custom made two-photon miniscopes were built as described previously (6, 7)), but featuring an improved scope body design made from machinable plastic (PEEK-CF30), which lowered the weight (2.6g) compared to previous versions (8). The supple fiber bundle (SFB) used in previous versions was substituted by a lighter and more flexible, 0.7 mm diameter fiber bundle with a tapered end piece (TFB), in which scattered fluorescence emerging from the sample is collected over a diameter of 1.5 mm and then coupled into the fiber bundle from the tapered end (8). A Ti:Sapphire laser (MaiTai Deepsee eHP DS, Spectra-Physics, repetition rate 80 MHz) tuned to a wavelength of 920 nm was used as the excitation source. Pulsed laser light was led through, in series, a half wave plate (HWP, AHWP05M-980, Thorlabs, NJ, USA) in combination with a polarizing beam splitter cube providing power attenuation, an electro-optic modulator (Model 350-80-LA-02, Conoptics, driver: 302RM) providing fast power modulation, and finally three 15-cm long ZF-62 glass tubes (GLA-10x150-AR800-1100, Sunlight, Fuzhou, China), followed by another HWP (AHWP05M-980, Thorlabs). The ZF-62 glass introduces constant positive dispersion, and the laser's internal dispersion compensation (Mai Tai Deepsee module) minimizes residual dispersion induced by the fiber. The laser light was coupled into a hollow-core photonic-crystal fiber (HC-920-01-FUD, batch 2015, NKT, Denmark) via an aspheric lens (f=11 mm, Geltech, C220TMD-B, Thorlabs) mounted on a fiber launch (MAX350D/M, Thorlabs). The coupling efficiency was monitored with a power meter (S121C connected to PM100D, Thorlabs) and small adjustments were made over time to maintain stable coupling. Coupling efficiency through the HC-PCF averaged around 80-85%.

Inside the scope body the laser was deflected by a MEMS scanner (A3I12.2-1200AL, Mirrorcle, CA, USA), before being led through a scan lens (D0131 / D0166, Domilight, Nanjing, China) and deflected by a dichroic mirror (HT 450-650nm, HR 800-1100nm, Sunlight Ltd., Fuzhou, Fujian, China) towards a custom designed mini objective (water-immersion, D0213, Domilight Ltd., Nanjing, China) and into the sample. Fluorescent light from the sample was led through the TFB into the detection module, consisting of an aspheric condenser lens (ACL25416U-A, Thorlabs) for collimation, low-pass filter (HC 720/SP, Semrock, AHF Analysentechnik AG Tuebingen, Germany), a dichroic beamsplitter plate (HC BS 560, Semrock, AHF Analysentechnik AG), followed by two transmission filters ("green channel": 525/50 BrightLine HC, Semrock; "red channel": 620/60 ET Bandpass, Chroma; AHF Analysentechnik AG), and two aspheric condenser lenses in each detection path (ACL25416U-A, Thorlabs) focusing the light on two GaAsP photomultiplier tubes (PMT2101, Thorlabs). A mechanical shutter (SHB1, Thorlabs) was put in front of the detection module to protect the PMTs from unexpected exposure. The MEMS driver (BDQ PicoAmp 5.4 T180, Mirrorcle, CA, USA) was mounted in a custom-made housing and connected to the MEMS scanner via a bundle of thin isolated wires.

The miniscope control stack consisted of NI hardware (NI PXIe-7961R NI FlexRIO FPGA Module, 3x PXIe-6341, X Series DAQ, NI 5734 Digitizer, chassis: NI PXIe-1073, BNC breakout boards: BNC-2090A) controlled via scanimage (9) (v.5.5, Vidrio Technologies, Virginia, USA). The MEMS scanner was driven in "resonant mode" in scanimage at 2 kHz resulting in a framerate of ~7.52 fps at 512x512 pixels. The average laser power before entering the GRIN lens was below 150 mW.

Miniscope imaging
At the beginning of every imaging session, mice were restrained through their headbar, with their limbs resting on a freely rotating treadmill. The treadmill was made from a ~85 by 70 mm (radius x width) styrofoam wheel with a metal shaft fixed through its center. Low friction ball bearings (HK 0608, Kulelager AS, Molde, NO) were affixed to the ends of the metal shaft and held in place on the optical table using a custom mount (https://github.com/kavli-ntnu/wheel_tracker). A table-top fluorescence microscope (SFM, Thorlabs), equipped with a 470 nm excitation source (M470L3, Thorlabs) and "GFP" excitation / emission / dichroic filter set (MDF-GFP2, Thorlabs) was used both to acquire epifluorescent images of chronic implants and to maneuver the two-photon miniscope over the mouse skull via a custom-made holder, which itself attached to the objective of the table-top microscope (MY5X-802-5X, Mitutoyo). The miniscope itself was held in place on the animal's head via a custom holder ("cup"), which was cemented in place above the implanted GRIN lens (with dental cement, see "GRIN / prism implantation" above), while imaging with the miniscope to determine the most suitable medio-lateral and anterior-posterior (=dorso-ventral through GRIN+prism) position. This procedure yielded a field-of-view (FOV) that was ideally both centered on MEC and had a high number of visible cells. For every subsequent recording the miniscope was inserted into the cemented cup in the same manner as during cementing / fixation, which enabled reliable re-finding of the FOV of previous recording sessions. The miniscope was then secured in place via two M1.6 set screws offset at 90 degrees in the holder base. After releasing the scope from its holder on the table-top microscope, two RGB LEDs (NeoPixel Nano, PID 3484, Adafruit, New York, USA) were mounted to the scope body on a light plexiglass bar (length: ~43 mm), which allowed tracking of both the animal's position in x and y and its head rotation in open field arenas. Mouse behavior in open field arenas was recorded in RGB via a camera mounted to the ceiling of the recording room (acA2040-55uc, Basler, Ahrensburg, Germany; objective: AZURE-1236ZM, RMA Electronics. Inc.). Brightness of the RGB LEDs (one set to only emit blue light, the other to only emit red light) was adjusted to enable smooth and reliable tracking via a custom written application that allowed both online tracking and visualization of animals' paths as well as parallel saving of recorded videos and extracted position data at 500x500 px and 40 Hz (https://github.com/kavli-ntnu/2P_tracking). Imaging and tracking were synchronised via TTLs (Frame clock of two-photon miniscope and exposure of new tracking frames (Basler camera GPIO interface)), which were recorded via WaveSurfer, an open-source software package for acquiring neurophysiology data in Matlab (https://wavesurfer.janelia.org/), on a NI PCIe-6321 card at 5 kHz. WaveSurfer triggered the miniscope acquisition and generated trigger pulses at regular intervals (40 Hz) for the tracking camera, which in turn sent TTLs for every exposure back to WaveSurfer.

Analysis pipeline
All analyses, from extraction of transients, deconvolution of fluorescence data (see below) to the analysis of spatial tuning and topographic properties were integrated in a custom pipeline using the Datajoint framework (https://datajoint.io/), an open-source library for programming scientific databases and computational data pipelines (10). The open-source package opexebo (https://pypi.org/project/opexebo/) was used to analyze all tuning properties, including field detection, grid score and border score calculations. Object vector cells were analyzed according to previously published methods (11) (see methods sections below).

Analysis of imaging time series
Two-channel imaging timeseries recorded via ScanImage (9) were analyzed using the Suite2p python library (https://github.com/MouseLand/suite2p), an open-source analysis pipeline for processing of two-photon calcium imaging data (12). We used its built-in motion correction, region of interest (ROI) extraction and spike deconvolution routines. For tdTomato cell analysis in SI Appendix, Fig. S4, cellpose (https://github.com/MouseLand/cellpose), an open-source generalist algorithm for cell and nucleus segmentation (13) was used on second channel average projections that were corrected by subtracting a linear offset extracted by regressing first against second channel projections for each session.

For calcium time series data, non-rigid motion correction was chosen to align each frame iteratively to a template and visual inspection of the corrected stacks and built-in motion correction metrics

were used to judge its quality. After automatic ROI extraction, the Suite2p GUI was used to manually sub-select putative neurons based on anatomical and signal characteristics and discard obvious artefacts that accumulated during the analysis (e.g., unrealistically large ROIs or ROIs that did not have a clearly delineated footprint in the generated maximum intensity projection). Raw fluorescence traces were processed to create a corrected fluorescence signal (F_{corr}) by first subtracting 0.7 * neuropil signal, and ΔF/F was then calculated as (F_{corr} - F_0) / F_0. F_0 was estimated as constant for each cell after smoothing F_{corr} with a 15 s spanning window (convolution of a scaled window with the signal, after introducing reflected copies of the signal at both ends so that transient parts are minimized in the beginning and end part of the output signal) and taking the median of the minimum 20% of this signal. Non-negative deconvolution (14) was used for deconvolving the neuropil and baseline corrected fluorescence (F_{corr}), yielding unfiltered, deconvolved events. After deconvolution we kept events (i.e., filtered events) that were larger than one standard deviation over the mean (calculated over all extracted events for that neuron) and filtered out cells that did not meet a signal-to-noise ratio (SNR) cutoff of 3.5. We used the deconvolved, filtered amplitudes (events) as input for all subsequent analyses (spatial tuning maps etc.).

For SNR calculation, noise statistics were extracted from the ΔF/F traces after filtering for episodes in which no deconvolved events were present (F_{noise}). These episodes had to lie at least 1 second before and 10 seconds after any deconvolved event to prevent signal contamination (safety margins). The SNR of that cell was then calculated as the ratio of the mean amplitude of filtered events over the standard deviation of F_{noise}. If no filtered events were maintained for a cell, a SNR of zero was assigned. Since the MEMS scanner of the two-photon miniscope introduces warping artefacts to the image due to inhomogeneities in its control voltage response, unwarping procedures were used to re-align ROI pixel and image projection data (i.e., average, or maximum intensity projections). For this, a standard grid distortion target (R1L3S3P, Thorlabs) was first imaged and piecewise affine transformation was used to re-align warped key points (crossings of all grid lines) to an idealized grid pattern. The resultant transformation matrix was then applied to unwarp both projection data (e.g., average, maximum-intensity projections) as well as ROI data. We filtered out ROIs that retained less than 10% of their original number of pixels after unwarping to get rid of edge artefacts for topographic analyses. Since the GRIN lenses we used have a magnification of 0.8 between the object plane and image plane), anatomical data that was acquired via these implants underwent additional post-correction with a scaling factor that was extract by aligning 2p miniscope FOVs to images acquired on the table-top fluorescence microscope through the same implant (see hardware description above, example can be seen in SI Appendix, Fig. S1B bottom).

Analysis of spatial tuning properties

Tracking (position of 2 LEDs on the mouse head) and imaging data (deconvolved, filtered fluorescence for each ROI) were synchronized by timestamp. As the tracking sample rate was ~5 times greater than that of imaging, each ROI could be assigned tracking data with greater temporal precision by assigning a small temporal offset to the ROI data between the start and end time of each imaging frame acquisition (~133 ms per frame), depending on the time it took the laser to reach that ROI within each FOV. Raw position and angular (head direction) data was smoothed with a Gaussian kernel (sigma 50 ms) and the instantaneous speed of the animal was then calculated. We applied speed cutoffs after smoothing the speed signal with a Gaussian kernel (sigma 150 ms, lower limit: 25 mm/s, upper limit: 500 mm/s) before analyzing any spatial tuning. Spatial data was binned (25 x 25 mm) and smoothed with a Gaussian kernel (sigma 50 mm). Basic occupancy statistics were used as quality markers, namely the exploration ratio ("ratio", how many bins of all bins were visited) and exploration standard deviation ("std", standard deviation of the time the animal spent across all bins). Higher exploration ratio and lower standard deviation together indicate more homogeneous exploration of the environment (Fig. 2 B and C and SI Appendix, Fig. S2A). Spatial tuning maps were created by dividing the binned imaging data (filtered, deconvolved fluorescence) by occupancy in each bin. Similarly, for head direction tuning analyses, the angular signal was smoothed with a Gaussian kernel both temporally and spatially (bin size 2 degrees, temporal smoothing sigma 4 degrees, spatial smoothing sigma 6 degrees). The open-source package opexebo (https://pypi.org/project/opexebo/) was used to analyze all tuning

properties from spatial tuning maps and angular tuning curves. Perceptually uniform sequential colormaps from the CMasher python library (15) (v1.6.1) were used for display of 2D tuning maps.

Field detection in spatial tuning maps (opexebo)
Place fields in spatial tuning maps and 2D autocorrelations (see Grid Score calculation) were identified by using an adaptive threshold method. First, local maxima (peaks) were found using SEP (16) (a python library for source extraction and photometry, https://pypi.org/project/sep/). Then, looping over every single peak, an initial threshold was determined (usually 0.8 of maximum) that, when lowering it in small increments, would yield an expansion of the field area around that peak. This field specific starting threshold was then systematically lowered in steps of 0.02 while field properties were measured until it reached one of several stopping criteria: 1. the field area could not be determined, 2. voids ("holes") appeared within field, 3. the expansion of the field lead to inclusion of other field peaks, 4. the field size more than doubled in one step or increased by the same or greater proportion in three consecutive steps, or 5. the field size did not change over 10 consecutive steps. Finally, fields were filtered out that had mean amplitudes below 10% of the global maximum or spanned fewer than 5 connected bins in the spatial tuning map.

Grid Score calculation (opexebo)
Spatial autocorrelograms of spatial tuning maps were used to obtain a measure of 60-degree place field periodicity (grid score). Peaks in spatial autocorrelations were found as described above ("Field detection in spatial tuning maps"). The grid score was calculated by expanding a circle around the center field and calculating a correlation value of that circle with rotated versions of itself. For each radius the score was calculated as the difference of the minimum correlation value at 60 and 120 and the maximum value at 30, 90, and 150 degrees. The final gridness score value was then obtained by calculating a maximum over a sliding mean of expanding circles. To determine grid spacing (distance of fields to center peak) and orientation, the orientation and distance of the six closest fields to the center peak was quantified. If two fields had less than 20 degrees difference from each other, the one with the larger distance from center was discarded. The first three fields were maintained, starting from the horizontal axis, and progressing in ascending (angular) order. The average orientation was extracted as the average difference to 60 degrees axes.

HD tuning curves and mean vector length
For circular statistics, we adapted scripts from the circstat toolbox (17) To obtain head direction tuning curves, filtered and deconvolved fluorescence was binned in 2 degree radial bins and divided by occupancy. Tuning curves were smoothed with a Gaussian kernel (sigma 6 degrees). The mean resultant vector length (mean vector length, MVL) for circular data was then calculated as described previously (18) and ranged from 0 to 1, where 1 is the case that all data are concentrated in the same direction. The calculation is described in (19), Section 26.4.

Border score (opexebo) and boundary vector score
The border score was calculated for spatial tuning maps as in (20). This score is in the range [-1, 1] and reflects both the width of a field (what fraction of a single wall it touches), and the depth of a field (how far away from the wall it extends). It was only evaluated for the single firing field that had the greatest wall coverage. All other fields were ignored. The highest scores are returned for cells that have a field both with maximum width along one wall of the square open field and shallow depth. Scores are usually below ~0.9 in a typical spatial tuning map (limited by the width of the binning compared to the arena extent). To account for additional firing fields, fields that had elongated fields parallel but offset from one of the walls, and those cases in which fields were less perfect (i.e., shallow) but still visibly border-like we calculated a boundary vector score (BVS). This score is in the range 0 to 1, where 1 is assigned to cells that have perfect and isolated border fields, which may or may not be offset from one of the walls. For this, the spatial tuning map of a cell was first binarized, with bins above median + 1 standard deviation over all bins set to one and zero for the rest. Then connected regions (=fields) were detected and quantified and those that covered fewer than 16 bins or had maximum amplitudes below the median + 2 standard deviations over all bins in the unprocessed spatial tuning map were discarded, thus yielding a filtered field map. Bars

of variable thickness (from 1 to 5 bins) spanning the whole width / height of the spatial tuning map were then moved over the map in x / y and at each position a score was calculated as: $\text{Overlap}_{\text{Bar}} - r * \text{Overlap}_{\text{Rest}}$, where $\text{Overlap}_{\text{Bar}}$ is the fraction of bins in the bar overlapping with a field and $\text{Overlap}_{\text{Rest}}$ is the fraction of bins overlapping with a field outside the bar. The r-value is a factor in range 0 to 1, which weighs the contribution of extra-bar field bins in decreasing the score and was set to 0.5 in this study. The maximum score over all different combinations of bar widths and position offsets was chosen as the boundary vector score for that cell. The BVS was preferred over the border score when a more graduated evaluation of border responses was needed (i.e., topographic tuning maps).

Shuffling distribution and cutoffs for spatial scores

Shuffled distributions of spatial scores were created for each cell by randomly shifting the tracking versus the deconvolved fluorescence signal in time. Data points that were shifted beyond the last element were reintroduced at the first. A total of 500 shuffle iterations were introduced per cell, with possible shifts being at least 0.5 seconds apart to account for the fact that due to slow time courses of the calcium sensor, neighboring timepoints could not be considered independent samples. A safety margin of 2 seconds was introduced at both ends of the timeline. Spatial scores were calculated for each shuffling iteration and each cell's real score was compared to percentile cutoffs of a session wide distribution (shuffling results over all filtered cells per session). Shuffling cutoffs used throughout the study were the 95th percentile of shuffled distributions unless otherwise indicated. When drawing pairwise comparisons between cell populations (cell type ratios in Fig. 3 and SI Appendix Fig. S3 and cell distances in Fig. 4 and SI Appendix Fig. S5), we classify cells as "pure" when they cross threshold criteria for only one of the two cell types under comparison and "conjunctive" if they cross more than one.

Object vector cells

Object vector cells (OVCs) were usually identified over three adjacent sessions (15-20 minutes each), comprising a base session without objects and two object sessions. For this three-session design, one object (a 64x64x230 mm colorful duplo brick tower) was placed in the middle of one of the open field quadrants in the first object session and moved to the middle of the diagonally opposite quadrant in the second object session. In rare cases only two sessions were run (one base and one object session) and similar looking objects were placed in opposing quadrants of the open field. To analyze object related firing patterns, object centered occupancy and tuning maps (object vector maps) were first created as described previously (11), by tiling space around the center of each object in 72 radial wedges, which were further subdivided along distance from the center into 2.5 cm wide bins. The object vector score (OV score) was then defined as the 2D correlation between the two object vector maps. Fields in the spatial tuning map were analyzed as described above ("Field detection in spatial tuning maps") and for every field, an object centered distance and angle was extracted, from the center of the object location to its centroid. Then, putatively matching fields in the spatial tuning map across objects (either two sessions with one object each or two objects in one session) were identified by calculating all pairwise Euclidean field distances in object centered coordinates and keeping the one with minimum distance to each other. To filter for OVCs, a range of cascading criteria were used: 1. OV scores had to be above the 95th percentile shuffling cutoff (evaluated on cellular, not session level), 2. Spatial tuning maps had to have information content above the 95th percentile shuffling cutoff in each of the object sessions (evaluated on cellular, not session level). Information content was calculated as described in (21) ([bits / deconvolved fluorescence]), 3. The maximum distance in object centered space between putatively matching fields in both object sessions had to be below 250 mm and they were required to have a minimum distance to the object of 40 mm, and finally 4. fields had to show at least 50% increase in activity compared to the same location in the baseline session. These criteria were used to ensure that putative OVCs developed clearly delineated firing fields in object sessions (information content and ratio of activity to base sessions) and that those fields were in a distance / angle relationship that matched expectations for OVCs (ensured by both OV score and distance cutoffs).

Grid module extraction
Example data is shown in SI Appendix, Fig. S8. We ran principal component analysis (PCA) on arrays of spacing versus orientation data from a subset of grid cells with high grid scores (>99th percentile shuffling distribution cutoff) in each animal. After dimensionality reduction, unsupervised clustering (HDBSCAN, https://github.com/scikit-learn-contrib/hdbscan) was run on this dataset of to extract seed clusters (i.e., preliminary grid modules; “skeleton”, SI Appendix, Fig. S8 A top). A k-nearest neighbor classifier (kNN implementation in scikit-learn (22), v0.24.2) was then trained on this data; this trained classifier was subsequently applied to expand each module by adding grid cells with >95th (and <99th) percentile shuffling cutoff (SI Appendix, Fig. S8 A bottom). Grid modules that contained fewer than 5 cells were discarded.

Session FOV alignment
Due to changes in the exact clamping angle and position of the miniscope in its holder (“cup”) from day to day, the field of view (FOV) had to be aligned to a reference before projections over multiple recordings could be created (described in detail below). The goal was to create alignments on the coarse macroscopic level, maintaining alignment of for example the blood vessel pattern and not necessarily on the (sub-)cellular level. Matching anatomical landmarks were picked in both the GCaMP (“green”) and tdTomato (“red”) channels for every pair of reference / session FOVs. The reference FOV was first picked, which showed maximum anatomical overlap with most other FOVs. Then landmarks were picked in a custom written software (PyQT5 GUI), which allowed inspection of both cellular details (average and maximum intensity projections) and blood vessel patterns (average intensity projections) in both channels. At least three pairs of points had to be defined for successful alignment (number of point pairs: (mean ± SD) 7±2). The reference was first padded and nudged to create sufficient space for the creation of a stitched composite. FOVs were then warped into a common coordinate space through an affine transformation defined over the user-defined point pairs. To evaluate the increase in anatomical overlap after versus before alignment of recorded FOVs, the structural similarity index (SSIM) (23) was calculated for same-sized areas in the average projections of aligned and reference session FOV, and compared before and after alignment. An increase in SSIM signified that the alignment yielded an improvement in anatomical overlap. The average projection was chosen because it maintained anatomical information (e.g., blood vessel patterns) while blurring cellular details that would otherwise disturb the alignment assessment.

FOV score map projections, Moran’s I and correlations
To obtain FOV score maps (Fig. 6 and SI Appendix, Fig. S7D and Fig. S8C), anatomical composites were first created as described above (“Session FOV alignment”) and the boundaries of each composite were used to create binning vectors (bin size 5 μm). Unwarped and aligned ROI pixel data was then binned in x and y (filtered session ROIs). Each 25 μm^2 bin was assigned a score defined as the maximum over all pixels inside that bin (e.g., max. grid score of all ROI pixels in each bin). Bins that did not contain any data in the first place were set to NaN. Since most spatial scores tend to not fall off smoothly at the lower end of their range (for example grid scores or border scores), pixels containing values below shuffling cutoff were set to zero. Unless otherwise indicated, the 95th percentile shuffling cutoff (session level) was used for grid, border and HD (MVL) scores and combined criteria as described above were used for object vector cells. The final FOV score maps were then obtained by taking the average projection of the aligned and binned score data of each session FOV. For border cells, the boundary vector score is plotted, for reasons indicated above, i.e., to obtain smoothness of values across the possible range of scores. To analyze spatial clustering in score maps, Moran’s I, a global autocorrelation statistic, was used (24). It is a measure of how similar neighboring bins in a 2D map are to each other and captures the intuition that spatial clustering introduces dependencies of value changes in bins over space compared to a “salt and pepper” organization in which no such dependencies can be observed. Moran’s I values were calculated via the ESDA library, an open-source python library for the exploratory analysis of spatial data (sub module of PySAL,(25)). To obtain a significance level and compare each Moran’s I value to a “salt and pepper” organization, shuffled score maps were created by permuting cell IDs across all cells for all sessions that were used in the original projection. Random samples of shuffled maps for each of the sessions in the original projection

were then combined and Moran's I values were calculated as for the real data. This process was repeated 5000 times to create a distribution of Moran's I values and extract 1st/5th and 95th/99th percentile cutoffs respectively. FOV score map projections with Moran's I values that were above the 95th percentile cutoff are called "clustered" since they deviate most strongly from a salt and pepper (i.e. random) organization obtainable from the same data. To determine the relationship of cell class territories over anatomical space, FOV score maps were first smoothed with a Gaussian (sigma 10 microns) and then the 2D correlation was calculated. The correlation value obtained from this comparison was compared to a shuffled distribution obtained by correlating randomized FOV score maps to each other. The procedure for obtaining randomized maps was identical to the one described above for Moran's I values. 10,000 comparisons were drawn, and 1st/5th and 95th/99th percentile cutoffs were determined from the resulting distributions. Correlation values below the 5th percentile cutoff were considered "anti-correlated" and those above the 95th percentile correlated. Perceptually uniform sequential colormaps from the CMasher python library (15) (v1.6.1) were used throughout.

Masking MEC and adjacent structures

In some mice that were implanted more medially than average and that were labeled with retrogradely transported AAVretro-CAG-tdTomato in hippocampus, a clear drop-off of tdTomato fluorescence could be observed in parts of the FOV, demarcating the boundary between MEC (high expression of tdTomato) and adjacent structures (i.e., parasubiculum PAS, low expression). We used a custom-written workflow built in napari (https://napari.org/) to paint in anatomical masks for MEC and adjacent structures. Where no tdTomato boundary was observable, we relied instead on Nissl-stained histology to infer the putative FOV position and subsequently the anatomical region that was most likely covered by the implant. Extracted masks were used to filter ROI data and label each cell by its anatomical region.

Nearest neighbor (NN) analyses

Intra-cell class nearest neighbor distances (Figs. 4, S5, 5 and S6) were calculated with the help of PySAL (25) (https://pysal.org/, v2.3.0) and pairwise distances and inter-cell class distances (KDTree for fast generalized N-point problems) with tools from scikit-learn (22) https://scikit-learn.org/, v0.24.2).

Shuffling of Nearest Neighbor (NN) analyses

Unless otherwise indicated, shuffled distributions were created over n=1000 iterations and the median over all shuffled values was used for any normalizations involving results of this shuffling. For inter-cell class distances (i.e., two populations A and B, Fig. 4 and SI Appendix, Fig. S5) size matched groups were first created by randomly picking groups of cells that had the same number as the smaller population (A or B) in each case. For example, if population A contained 50 cells above threshold (starter cells), and population B 25 cells, A was subsampled to only contain 25 cells. This picking procedure was repeated 1000 times and the median over all permutation results was used for further calculations. Then, two population statistics could be extracted, i.e., reaching from class A to class B or class B to class A. In the idealized case that half of the FOV was covered by one or the other cell population, the directionality did not have an effect. However, because of FOV edge effects, differences could be quite pronounced. To not get biased towards either side, we took the average value of over those two statistics.

Graph analysis and spring-loaded model

For graph analysis in Fig. 4E we used an implementation of an undirected graph that can hold multiple nodes from the open source networkx python library (https://networkx.org/, v2.5). Weights between nodes (different cell classes) were assigned by the inverse of the mean over the nearest neighbor distances between two cell classes (see also "Nearest neighbor (NN) analysis" above) and were centered around zero. To introduce (random) variability before running the simulation, node positions were randomly initialized in a square centered on (0,0). The simulation itself utilizes the networkx implementation of the Fruchterman-Reingold algorithm (networkx.drawing.layout.spring_layout). This algorithm simulates a force-directed representation of the network, treating edges as springs holding nodes close, while trying to reach an equilibrium

state at every step at the simulation by re-arranging node positions according to the weight of their edges. The simulation was run for 1000 steps for Fig. 4E and SI Appendix, Fig. S5D but varied systematically in SI Appendix, Fig. S5E. To quantify the divergence of the “Grid” (grid cell) node compared to all other nodes, compensating for the varying distances that could occur in the simulation, we calculated the distance ratio of grid to all other cell nodes over the mutual distance of all other cell nodes (i.e., border, HD and OV nodes). The distance of grid to all other nodes was quantified as the Euclidean distance between the grid cell node and the center of all other nodes, and the mutual distance of all other (non-grid) nodes as the mean of pairwise distances between those nodes. Ratio values close to 1 indicate that the divergence between grid to all other nodes was similar to the distances in between all other (non-grid) nodes.

Statistical analyses

The details of statistical testing, including the test statistic, p-values, number of results and what the number of results refer to can be found in the figure legends throughout. Definition of distribution centers and dispersion for each Fig. are indicated in the Fig. legends and described in the main text. Randomization procedures are described in detail in the associated sections in the Method details section and explained in the main text and Fig. legends where appropriate. Most statistical testing was performed using non-parametric tests because Gaussian sample distributions could not be ascertained due to low sample numbers. All test statistics and p-values were calculated using SciPy’s stats module (v1.5.4) (26) and a significance level of 0.05 was used throughout to assess whether results were statistically significant ($p < 0.05$) or not ($p \geq 0.05$).

Figs. S1-S8

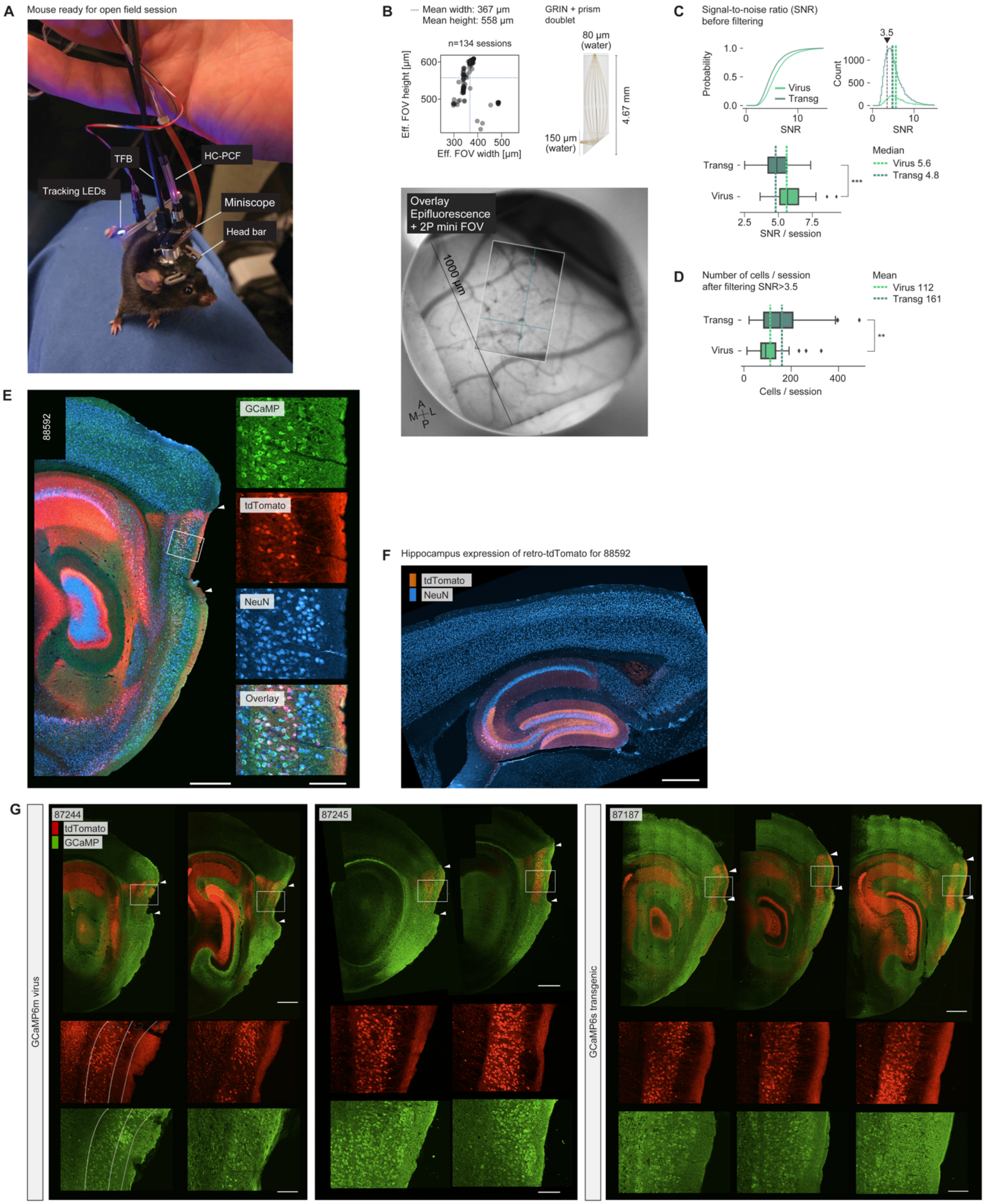

A
Mouse ready for open field session
TFB
HC-PCF
Tracking LEDs
Miniscope
Head bar
B
Mean width: 367 µm
Mean height: 558 µm
GRIN + prism doublet
n=134 sessions
80 µm (water)
4.67 mm
150 µm (water)
Eff. FOV height [µm]
Eff. FOV width [µm]
Overlay
Epifluorescence
+ 2P mini FOV
1000 µm
C
Signal-to-noise ratio (SNR) before filtering
Probability
Count
Virus
Transg
SNR
3.5
Median
Virus 5.6
Transg 4.8
SNR / session
D
Number of cells / session after filtering SNR>3.5
Mean
Virus 112
Transg 161
Cells / session
E
88592
GCaMP
tdTomato
NeuN
Overlay
F
Hippocampus expression of retro-tdTomato for 88592
tdTomato
NeuN
G
87244
tdTomato
GCaMP
87245
87187
GCaMP6m virus
GCaMP6s transgenic

Fig. S1. 2P miniscope, field of view (FOV), signal characteristics and example histology.

(A) Photo of a mouse carrying the 2P miniscope with annotations (TFB: Tapered fiber bundle (signal detection); HC-PCF: hollow-core photonic crystal fiber). The weight of all components mounted on the animal head was counterbalanced by a pulley system while animals were running inside the open field arena.

(B) Left: Effective (Eff.) FOV height and width (after motion correction) of 134 recorded sessions. Stippled blue lines indicate the mean (Mean width ± SD [µm]: 367 ± 40, Mean height ± SD [µm]: 558 ± 50 µm).

Right: Schematic of custom GRIN+Prism implant and its optical properties (Image side working distance: 80 µm in water; object side working distance 150 µm in water). Bottom: Overlay of two-photon imaging FOV (rectangle in center, time-series average projection) and epifluorescence image (whole image) through the same implant in vivo.

(C) Comparison of signal-to-noise ratio (SNR) across GCaMP6 transgenic ("Transg") and virus ("Virus") injected animals; n animals: 12 transgenic; 3 virus. Top: Distribution plots (left and right) across all cells. Colored, stippled lines indicate median; grey stippled line indicates SNR cutoff (3.5) used throughout this study to filter cells. Bottom: SNR per session. Box shows range from first to third quartile, whiskers indicate first / third quartile ± 1.5 times the inter quartile range (IQR), thick vertical line indicates median. The median SNR of cells recorded in transgenic animals was slightly lower than in virus injected animals (Median: Transg 4.80, Virus 5.61; Mann-Whitney U=844, n_{Transg}=103, n_{Virus}=31, p=0.000073***, two-sided).

(D) Comparison of number of cells per session across GCaMP6 transgenic ("Transg") and virus ("Virus") injected animals after filtering; n animals: 12 transgenic; 3 virus (Mean±SD [n cells]: Transg 160.7±96, Virus 111.5±68; Mann-Whitney U=1071, n_{Transg}=102, n_{Virus}=31, p=0.0033** two-sided). Box plot characteristics as in (C).

(E) Example immunofluorescence in a brain slice of a GCaMP6 transgenic mouse (same animal as in Fig.1C). White arrow tips indicate the dorsal end of MEC (top) and ventral edge of the prism (bottom). Insets to the right show magnification of the white box. GCaMP (green), tdTomato (orange), NeuN (cyan) and overlay (GCaMP + tdTomato + NeuN) are shown separately. While GCaMP positive cells are found across several layers, tdTomato expression is mainly restricted to layer III. Scale bar 500 µm, insets: 125 µm.

(F) Confocal image of a stained, sagittal brain slice of the animal shown in (E) and Fig. 1C, which shows expression of AAVretro-CAG-tdTomato in DG and CA3 of hippocampus, close to the injection site. Red: tdTomato, native fluorescence; blue: primary mouse aNeuN + 647 nm secondary. Scale bar 500 µm.

(G) Example sagittal slices of 3 animals with high fractions of functional cell types. Insets at the bottom of each image are magnifications of the white boxed regions. Green: GCaMP6 (aGFP), red: retro-tdTomato (native fluorescence). GCaMP virus injected animals: 87244, 87245; GCaMP transgenic animal: 87187. Dense cellular labeling was observed in the superficial layers of MEC in all animals. White arrow tips indicate the dorsal end of MEC (top) and most ventral imprint of prism (bottom). Depression of the brain surface surrounding the white box reflects the location of the prism. An example layer annotation (Layer 1 to 3) is shown for animal 87244 (left). Scale bars 500 µm, insets: 125 µm. Additional pictures of GCaMP and tdTomato expression in sagittal brain slices are shown for animal 88592 (GCaMP transgenic animal) in SI Appendix, Fig. S4 (top) and animal 88106 (GCaMP transgenic animal) in SI Appendix, Fig. S4 (bottom).

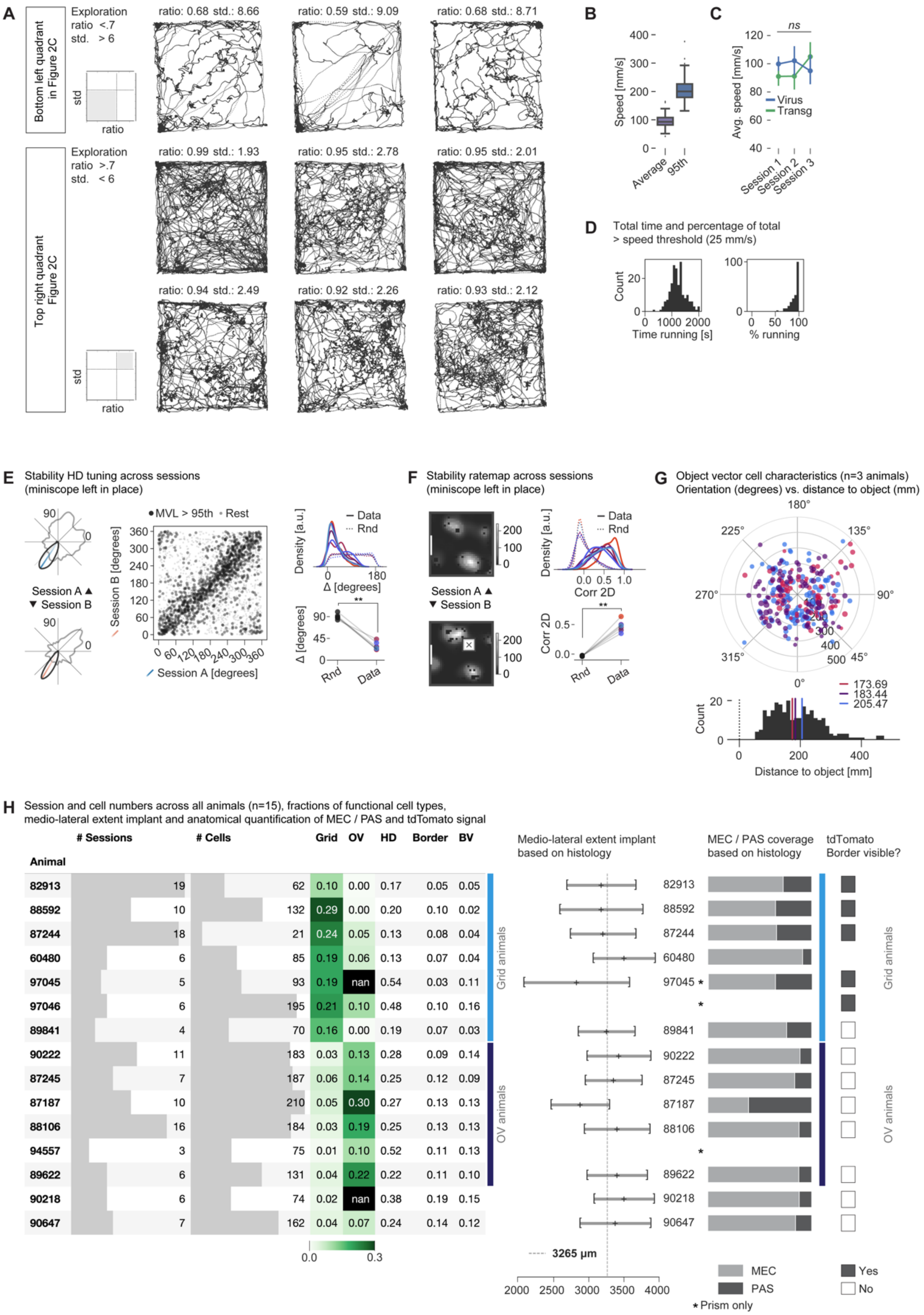
A
Bottom left quadrant in Figure 2C
Exploration ratio <.7 std. > 6
std
ratio
ratio: 0.68 std.: 8.66
ratio: 0.59 std.: 9.09
ratio: 0.68 std.: 8.71
Top right quadrant Figure 2C
Exploration ratio >.7 std. < 6
ratio: 0.99 std.: 1.93
ratio: 0.95 std.: 2.78
ratio: 0.95 std.: 2.01
ratio: 0.94 std.: 2.49
ratio: 0.92 std.: 2.26
ratio: 0.93 std.: 2.12
B
Speed [mm/s]
Average
95th
C
ns
Avg. speed [mm/s]
Virus
Transg
Session 1
Session 2
Session 3
D
Total time and percentage of total > speed threshold (25 mm/s)
Count
Time running [s]
% running
E
Stability HD tuning across sessions (miniscope left in place)
Session A ▲
▼ Session B
MVL > 95th
Rest
Session B [degrees]
Session A [degrees]
Density [a.u.]
Data
Rnd
Δ [degrees]
F
Stability ratemap across sessions (miniscope left in place)
Session A ▲
▼ Session B
Density [a.u.]
Corr 2D
Data
Rnd
G
Object vector cell characteristics (n=3 animals)
Orientation (degrees) vs. distance to object (mm)
173.69
183.44
205.47
Count
Distance to object [mm]
H
Session and cell numbers across all animals (n=15), fractions of functional cell types, medio-lateral extent implant and anatomical quantification of MEC / PAS and tdTomato signal
Animal | # Sessions | # Cells | Grid | OV | HD | Border | BV
82913 | 19 | 62 | 0.10 | 0.00 | 0.17 | 0.05 | 0.05
88592 | 10 | 132 | 0.29 | 0.00 | 0.20 | 0.10 | 0.02
87244 | 18 | 21 | 0.24 | 0.05 | 0.13 | 0.08 | 0.04
60480 | 6 | 85 | 0.19 | 0.06 | 0.13 | 0.07 | 0.04
97045 | 5 | 93 | 0.19 | nan | 0.54 | 0.03 | 0.11
97046 | 6 | 195 | 0.21 | 0.10 | 0.48 | 0.10 | 0.16
89841 | 4 | 70 | 0.16 | 0.00 | 0.19 | 0.07 | 0.03
90222 | 11 | 183 | 0.03 | 0.13 | 0.28 | 0.09 | 0.14
87245 | 7 | 187 | 0.06 | 0.14 | 0.25 | 0.12 | 0.09
87187 | 10 | 210 | 0.05 | 0.30 | 0.27 | 0.13 | 0.13
88106 | 16 | 184 | 0.03 | 0.19 | 0.25 | 0.13 | 0.13
94557 | 3 | 75 | 0.01 | 0.10 | 0.52 | 0.11 | 0.13
89622 | 6 | 131 | 0.04 | 0.22 | 0.22 | 0.11 | 0.10
90218 | 6 | 74 | 0.02 | nan | 0.38 | 0.19 | 0.15
90647 | 7 | 162 | 0.04 | 0.07 | 0.24 | 0.14 | 0.12
Grid animals
OV animals
Medio-lateral extent implant based on histology
3265 µm
MEC / PAS coverage based on histology
MEC
PAS
tdTomato Border visible?
Yes
No
* Prism only

Fig. S2. Behavior, tuning stability, OV cell characteristics and summary of recorded cells and implant locations.

(A) Example path plots of sessions in the bottom left (top) and upper right (bottom) quadrants in Fig. 2C. Exploration ratio ("ratio") and standard deviation ("std") values are shown above each plot.
(B) Speed comparisons (same data as analyzed for Fig. 2C). Left: Average and 95th percentile (95th) of speed distributions across recording sessions; Average speed ± SD: 94.7 mm/s ± 20.6; 95th percentile speed ± SD: 203.7 mm/s ± 37.5.
(C) Speed across multiple, consecutively run sessions (<60 seconds break between adjacent sessions) shows that animal behavior stayed constant (e.g., across an open field and two object sessions) (figure shows mean ± 99th CI; Kruskal-Wallis H=3.68, $n_{Session1}$=123, $n_{Session2}$=48, $n_{Session3}$=32, p=0.159 ns); ns, not significant (p>0.05); virus injected WT animals: blue; transgenic GCaMP animals: green.
(D) Distribution of time spent running, i.e., above the speed threshold of 25 mm/s (left, absolute; right, relative to whole session length) for recordings shown in Fig. 2C; time: 1304.8 s ± 479.8 (mean ± SD); percentage of whole session spent running: 91.5% ± 8.8 (mean ± SD).
(E+F) Stability of single cell head direction (HD) tuning curves and spatial tuning maps across sessions. Tuning stability from one session to the next (session "A" and "B"; miniscope not detached between sessions, the animal is sometimes taken out of the box, handled, and put back in).
(E) Tuning stability of head direction cells recorded in two sessions. Left: Tuning curves of one cell recorded in two consecutive open field sessions ("A" and "B"). Blue and orange lines show (approximate) average angle of the tuning curve. Scatter plot in the middle shows average tuning angle in Session A vs. B for 2,615 cells across 6 animals with thick black dots indicating cells that met the 95th percentile shuffling cutoff (1,112 cells > MVL 95th). Dots correspond to individual cells. Right: Absolute difference in degrees of tuning angle in session A vs. session B (Data, colors indicate animals, n=6), compared to randomly (Rnd) picked cell pairs. All cells filtered by MVL > 95th percentile. Top: Kernel density estimate, bottom: Median per animal. The median tuning angle difference is significantly lower for analyzed cell pairs, compared to random, co-recorded cell pairs (Mann-Whitney U=0, n_{Data}=6, n_{Rnd}=6, p=0.0025**, two-sided). Note that the Gaussian kernel used in the density estimate (top) introduces artificial smoothing at the edges of the data range (0 and 180 degrees).
(F) Tuning stability of cells with high information content (>95th shuffling cutoff for information content). Left: Spatial tuning maps of one cell recorded in two adjacent sessions (session "B" is an object session, object location indicated by cross in white box). Analysis as in (E), but with spatial tuning map correlation values instead of tuning angle difference (Mann-Whitney U=0, n_{Data}=6, n_{Rnd}=6, p=0.0025**, two-sided).
(G) Distribution of field orientation and distances to objects in OV cells match previously published results with tetrodes (colors=animals, n=3 animals, 260 cells). Bottom: Histogram of field-to-object distances: dashed line indicates 0, vertical-colored lines indicate the mean distance to objects over all fields for each animal.
(H) Left: Number of sessions (# Sessions) and mean number of cells (# Cells, after filtering) per animal. Size of the grey bar in the background scales with the absolute value in each table entry. Next, the mean fraction (compared to all filtered cells in a recording) of grid, object vector (OV), head direction (HD), border and boundary vector (BV) cells per session (> 95th percentile shuffling cutoff) are shown. Grid and OV fractions are color coded (colorbar is shown at the bottom). Animals are ordered by "Grid animals" (blue bar, ≥ 10% grid cells on average), "OV animals" (violet bar, ≥ 10% OV cells on average) etc. Middle: Implant positions across the 13 animals analyzed in this study. Sagittal, Nissl-stained brain slices were aligned to the Allen mouse brain common coordinate framework (CCFv3) and implant covered regions were manually annotated. The line chart in the middle shows the medial to lateral extent of the implant, with the '+' denoting the midpoint. The dashed vertical line (light grey) indicates the average across animals (~3.3 mm lateral from midline). The horizontal bar plots towards the right indicate the approximate ratio between the volume covered by MEC (light grey) vs. PAS (dark grey). Grid animals were on average implanted more medially compared to OV animals. Right: Indication of whether a clear border was visible in the retro-tdTomato signal - indicating the transition between MEC (lateral) and PAS (medial) - either at the single session level or after stitching all session FOVs together (dark grey - yes, empty box -

no). Where no box is shown, no retro-tdTomato was injected. This medio-lateral region boundary was visible in most grid animals injected with retro-tdTomato.

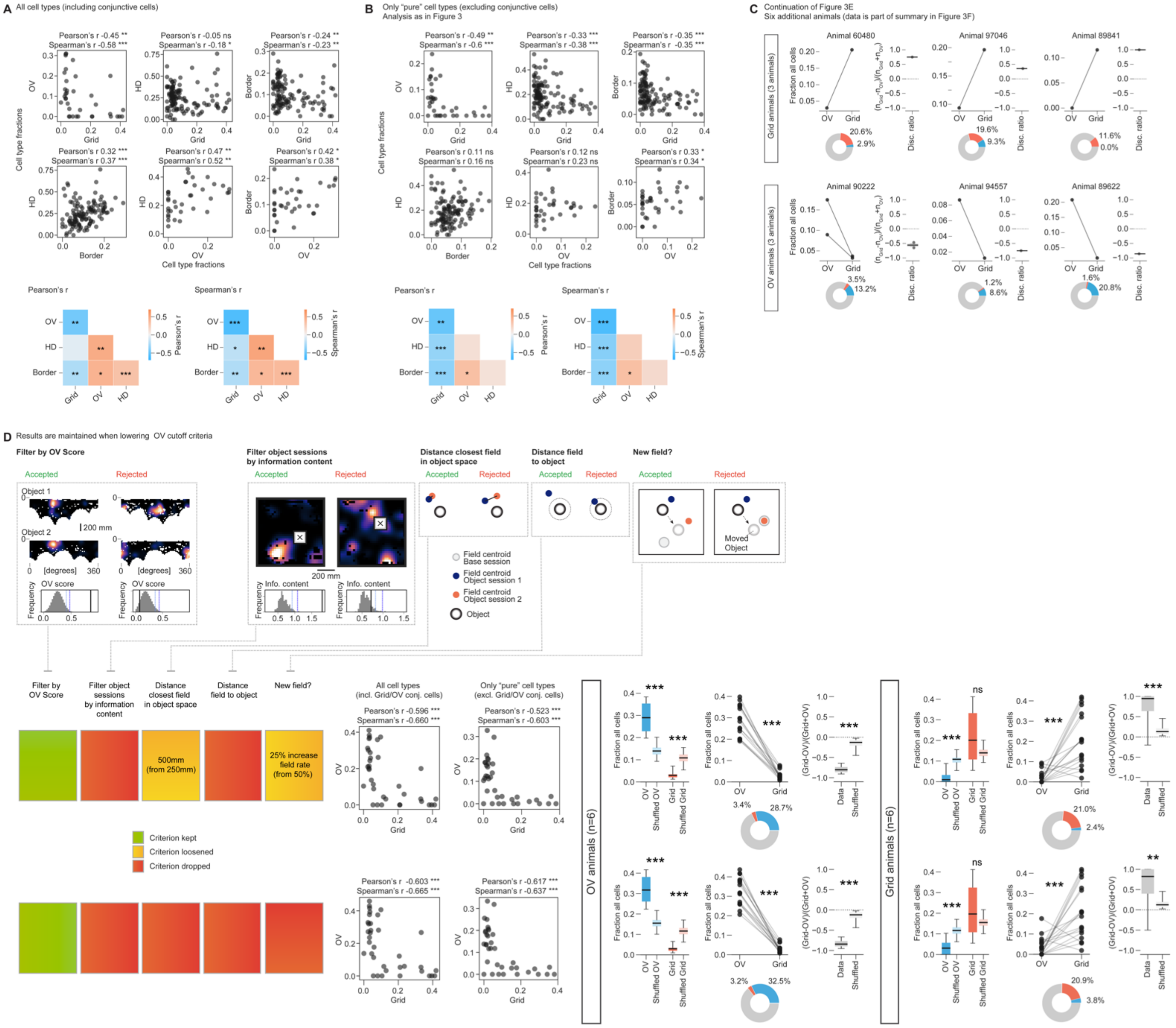

Fig. S3. Co-occurrence degrees of different cell types and effect of relaxing criteria for OV cells.

(A) Cell type fractions across all cells per session (comparison as in Fig. 3C; each dot represents one session). Conjunctive cells, i.e., cells that cross the threshold for more than one cell class (95th percentile shuffling cutoff for Grid, HD, Border and combined criteria for OV), are included. Pearson's and Spearman's correlation (r values) and significance tests are indicated in the top right corner of each scatter plot (15 animals, 124 sessions, filtered by MEC, minimum cutoff of 15 cells after filtering). Heatmap on the bottom shows summary of correlation values shown in top graphs. Pearson's correlation: Grid vs. Border: r=-0.24, $n_{Grid}=n_{Border}=124$, p=0.008**; Grid vs. HD: r=-0.05, $n_{Grid}=n_{HD}=124$ p=0.55ns; Grid vs. OV: r=-0.45, $n_{Grid}=n_{OV}=36$, p=0.0054**; OV vs. Border: r=0.42, $n_{OV}=n_{Border}=36$, p=0.011*; OV vs. HD: r=0.47, $n_{OV}=n_{HD}=36$, p=0.0038**; HD vs. Border: r=0.32, $n_{HD}=n_{Border}=124$, p=0.0003***. Spearman's correlation: Grid vs. Border: r=-0.23, $n_{Grid}=n_{Border}=124$, p=0.0093**; Grid vs. HD: r=-0.18, $n_{Grid}=n_{HD}=124$, p=0.041*; Grid vs. OV: r=-0.58, $n_{Grid}=n_{OV}=36$, p=0.0002***; OV vs. Border: r=0.38, $n_{OV}=n_{Border}=36$, p=0.021*; OV vs. HD: r=0.52, $n_{OV}=n_{HD}=36$, p=0.0012**; HD vs. Border: r=0.37, $n_{HD}=n_{Border}=124$, p= 2.37e-05***; ns not significant (p>0.05).

(B) Same as in (A) but excluding conjunctive cells (same as in Fig. 3C and D). Pearson's correlation: Grid vs. Border: r=-0.35, $n_{Grid}=n_{Border}=124$, p=5.32e-05***; Grid vs. HD: r=-0.33, $n_{Grid}=n_{HD}=124$, p=0.00017***; Grid vs. OV: r=-0.49, $n_{Grid}=n_{OV}=36$, p=0.00240**; OV vs. Border: r=0.33, $n_{OV}=n_{Border}=36$, p=0.048*; OV vs. HD: r=0.12, $n_{OV}=n_{HD}=36$, p=0.49 ns; HD vs. Border: r=0.11, $n_{HD}=n_{Border}=124$, p=0.23 ns. Spearman's correlation: Grid vs. Border: r=-0.35, $n_{Grid}=n_{Border}=124$, p=5.47e-05***; Grid vs. HD: r=-0.38, $n_{Grid}=n_{HD}=124$ p=1.27e-05***; Grid vs. OV: r=-0.596, $n_{Grid}=n_{OV}=36$, p=0.00013***; OV vs. Border: r=0.34, $n_{OV}=n_{Border}=36$, p=0.046*; OV vs. HD: r=0.23, $n_{OV}=n_{HD}=36$, p=0.17 ns; HD vs. Border: r=0.16, $n_{HD}=n_{Border}=124$, p=0.084 ns; ns not significant (p>0.05).

(C) Visualization as in Fig. 3E. Fraction of (pure) grid and OV cells and discrimination index over grid and OV cells in 6 additional animals (included in the summary in Fig. 3F), with higher fractions of grid cells than OV cells (top row, "Grid animals") or the opposite (bottom row, "OV animals"). Pie charts underneath each line plot show the average percentage of grid and OV cells across sessions. Boxes in box plots extend from lower to upper quartile values of the data, with a vertical line indicating the population median, whiskers indicate first to 99th percentile. A dashed line is drawn at 0.

(D) Lowering cutoff criteria for OV cell selection does not change the obtained result.

Top row: Schematic of the OV cell selection criteria (as described in SI Appendix, Extended Methods), from left to right: Filtering by object vector score (OV score), information content in object sessions, and field filtering criteria, i.e., distance of closest field in object space (inter field distance), distance of field to object, increase of activity rate of object related fields in comparison to baseline activity rate. Filtering by OV score and information content show example cells (accepted and rejected cases, scale bar 200 mm) and shuffling distributions (OV score and information content shuffling in grey, data indicated by black line, 95th and 99th percentile bright and dark blue stippled lines respectively, 500 shuffling iterations per cell). 95th percentile cutoffs are used throughout.

Bottom row: Two different cutoff methods, resulting in data shown on the right. Green: criterion kept, yellow: criterion changed, red: criterion dropped; Top: OV score 95th percentile cutoff unchanged, information content filter dropped, inter field distance threshold loosened (from 250 mm to 500 mm), distance threshold of field to object dropped, cutoff increase in activity rate loosened to 25% (instead of 50%). Bottom: All criteria dropped except for OV score filtering (green). Scatter plots show data as analyzed in panel (A) and (B) above. Data analysis on the right is analogue to summary data shown in Fig. 3E and F. Stars ('*') indicate significance level (statistical tests equivalent to those used in Fig. 3E and F): * p<0.05, ** p<0.01, *** p<0.001.

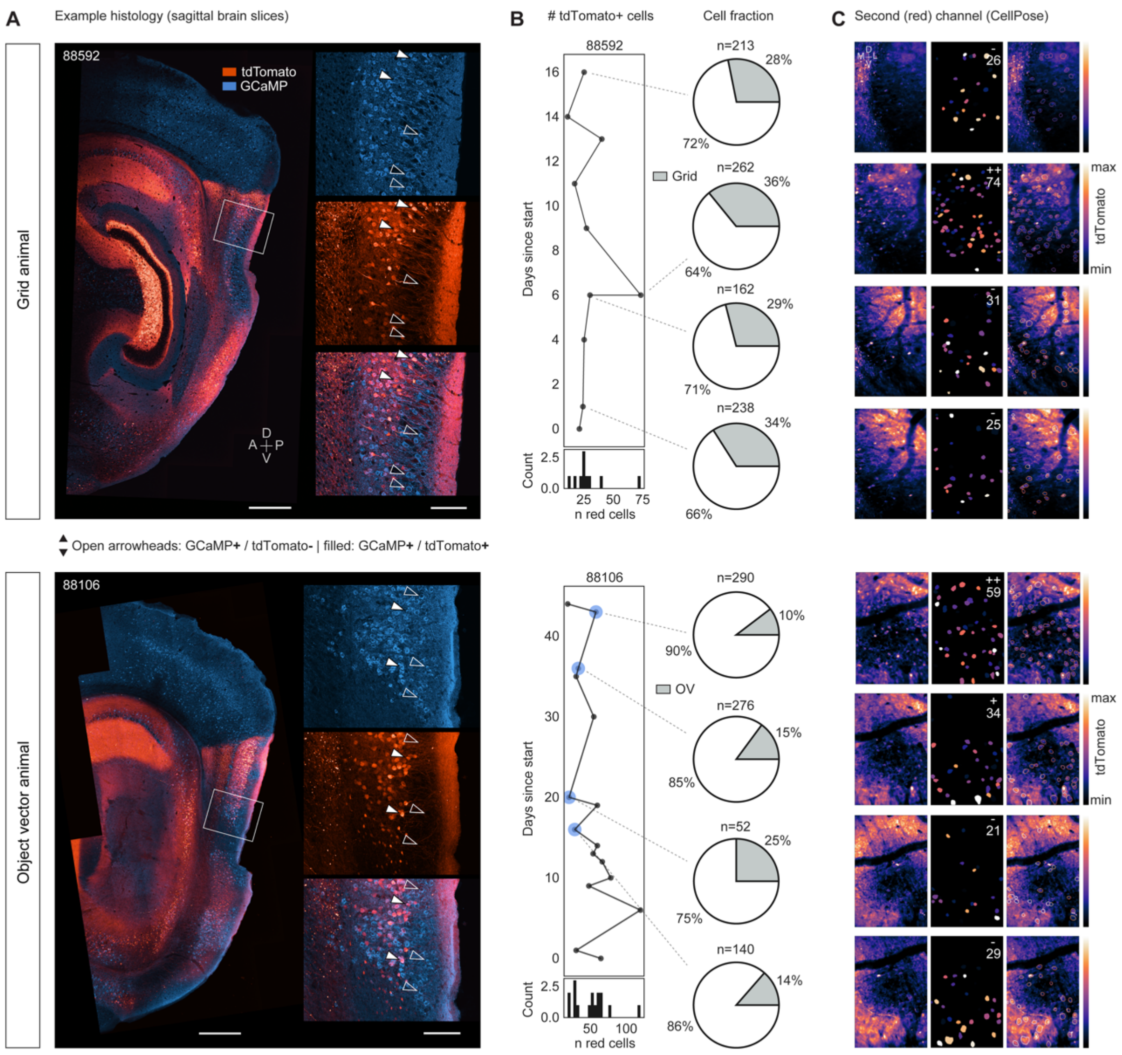

Fig. S4. Quantification of tdTomato positive cells in relation to the fraction of grid and OV cells in two example animals.

(A) Left: Confocal images of a sagittal brain slice in two example animals (blue: GCaMP6 (aGFP), red: tdTomato (native fluorescence)). Animal 88592 (top) had multiple grid cells per session ("Grid animal"), while 88106 (bottom) had multiple OV, but hardly any grid cells ("OV animal"). Insets show regions of MEC where the impression of the GRIN+prism implant is visible (approximate location of in vivo imaging). Arrow heads in insets point to GCaMP + tdTomato double positive cells (filled arrowheads) and GCaMP only labeled cells (unfilled arrowheads). TdTomato positive cells in these two example animals are enriched in layer III as opposed to layer II. See also SI Appendix Fig. S1G for histology in 3 additional animals injected with retro-tdTomato. Counting cells in the secondary (red) imaging channel thereby gives an indication of whether imaging was preferentially targeting layer III or II in each recording. Scale bars: 500 µm, insets: 125 µm.

(B) Timeline of recorded imaging sessions (days since start of imaging in each animal) and number of recorded red (tdTomato+) cells. The distribution across all recording sessions is shown on the bottom of each timeline. Pie charts towards the right highlight four example sessions for which the percentage of grid (top, 95th shuffling cutoff), and OV (bottom) cells is shown (blue circles indicate object sessions, total number of cells (n) shown on top of each pie chart). No apparent relation between recording date / number of red cells (i.e., cortical layer) and the fraction of grid / OV cells is visible.

(C) Quantification of red cells for the four example sessions indicated in the middle. CellPose was used to extract tdTomato+ cells from bleedthrough corrected average projections of the second (red) channel (see also SI Appendix, Extended Methods). Left column: Average projection. Middle column: CellPose result (cell masks, number of detected cells is indicated in top right; -: low, +: high, ++: highest). Right column: overlay of cell mask outlines on average projection of second (red) channel, colorbar indicates values in average projection (minimum to maximum).

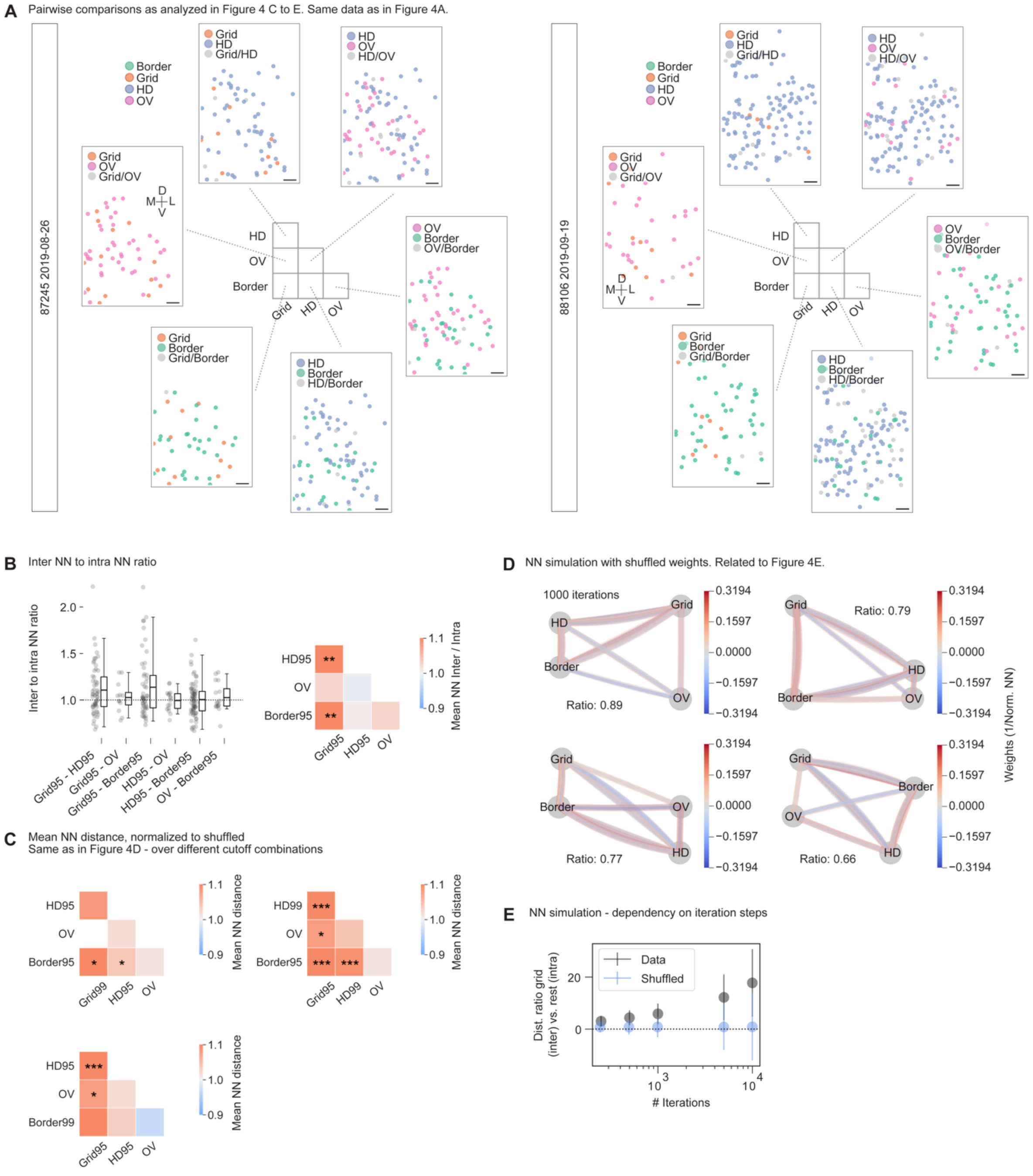

Fig. S5. Extended data for anatomical distribution of functional cell types in MEC.

(A) Example cell maps (same data as shown in Fig. 4A) of two recordings in animal 87245 (left, 322 cells) and 88106 (right, 309 cells), showing the distribution of border (green), grid (orange), HD (blue), and OV (pink) cells in pairwise combination (i.e., as analyzed in Fig. 4C to E). Cells are color labeled if they crossed shuffling cutoff criteria for one of the two cell types in each comparison and light grey if they met both cutoffs (the latter were excluded from the distance statistics). Tissue orientation is indicated by cross (D-dorsal, L-lateral, V-ventral and M-medial), Scale bars 50 µm.

(B) Inter to intra NN ratio measurements across sessions. Session and cell filtering as in Fig. 4D. The ratios of average inter-cell class distances (first nearest neighbors, as in Fig. 4B to D) over

intra-cell class distances (first nearest neighbors) were analyzed in size matched populations. Ratios above one indicate that cells were on average farther apart in between cell classes than within a cell class. The trends are in line with normalized NN distances shown in Fig. 4D. Left: Ratios across sessions. Kruskal-Wallis H=5.451, n=6 groups, p=0.36 ns; Right: Color coded average of results on the left. Stars indicate results of two-sided one sample t-test against population mean of 1 (95 indicates the 95th percentile shuffling cutoff used as inclusion criterion; p<0.05 *, p<0.01 **, p<0.001 ***; Grid/HD: 12 animals, mean ± SD: 1.11±0.27, t=2.90, $n_{Grid/HD}$=56, p=0.0054**; Grid/OV: 5 animals, mean ± SD: 1.03±0.16, t=0.567, $n_{Grid/OV}$=12, p=0.58 ns; Grid/Border: 12 animals, mean ± SD: 1.14±0.33, t=2.946, $n_{Grid/Border}$=51, p=0.0049**; HD/Border: 13 animals, mean ± SD: 1.01±0.19, t=0.226, $n_{HD/Border}$=68, p=0.82 ns; HD/OV: 6 animals, mean ± SD: 0.99±0.11, t=-0.19, $n_{HD/OV}$=16, p=0.85 ns; OV/Border: 7 animals, mean ± SD: 1.03±0.15 t=0.675, $n_{OV/Border}$=16, p=0.51 ns); ns: not significant (p>0.05).

(C) Same as in Fig. 4D (right), but over a different combination of cell class cutoff criteria (95: 95th percentile shuffling cutoff, 99: 99th percentile shuffling cutoff). Stars indicate results of two-sided one sample t-test against population mean of 1 (p<0.05 *, p<0.01 **, p<0.001 ***).

Top left: Changed Grid 95th to 99th percentile shuffling cutoff; Grid/HD: 7 animals, mean ± SD: 1.09±0.20, t=1.900, $n_{Grid99/HD95}$=21, p=0.072 ns; Grid/OV: 1 animal (white square in plot); Grid/Border: 6 animals, mean ± SD: 1.23±0.32, t=2.854, $n_{Grid99/Border95}$=17, p=0.011*; HD/Border: 13 animals, mean ± SD: 1.04±0.15, t=2.260, $n_{HD95/Border95}$=68, p=0.027*; HD/OV: 6 animals, mean ± SD: 1.02±0.07, t=1.065, $n_{HD95/OV}$=16, p=0.30 ns; OV/Border: 7 animals, mean ± SD: 1.01±0.13, t=0.395, $n_{OV/Border95}$=16, p=0.7 ns;

Top right: Changed HD 95th to 99th percentile shuffling cutoff; Grid/HD: 12 animals, mean ± SD: 1.12±0.19, t=4.610, $n_{Grid95/HD99}$=52, p=2.73e-05***; Grid/OV: 5 animals, mean ± SD: 1.08±0.12, t=2.211, $n_{Grid95/OV}$=12, p=0.049*; Grid/Border: 12 animals, mean ± SD: 1.15±0.24, t=4.324, $n_{Grid95/Border95}$=51, p=7.31e-05***; HD/Border: 12 animals, mean ± SD: 1.09±0.19, t=3.671, $n_{HD99/Border95}$=58, p=0.00053***; HD/OV: 6 animals, mean ± SD: 1.04±0.13, t=1.266, $n_{HD99/OV}$=15, p=0.23 ns; OV/Border: 7 animals, mean ± SD: 1.01±0.13, t=0.395, $n_{OV/Border95}$=16, p=0.7 ns;

Bottom left: Changed Border 95th to 99th percentile shuffling cutoff; Grid/HD: 12 animals, mean ± SD: 1.13±0.18, t=5.364, $n_{Grid95/HD95}$=56, p=1.67e-06***; Grid/OV: 5 animals, mean ± SD: 1.08±0.12, t=2.211, $n_{Grid95/OV}$=12, p=0.049*; Grid/Border: 7 animals, mean ± SD: 1.14±0.34, t=1.996, $n_{Grid95/Border99}$=24, p=0.058 ns; HD/Border: 7 animals, mean ± SD: 1.03±0.08, t=1.628, $n_{HD95/Border99}$=19, p=0.12 ns; HD/OV: 6 animals, mean ± SD: 1.02±0.07, t=1.065, $n_{HD95/OV}$=16, p=0.3 ns; OV/Border: 4 animals, mean ± SD: 0.97±0.08, t=-1.088, $n_{OV/Border99}$=8, p=0.31 ns; ns: not significant (p>0.05).

(D) Analysis as in Fig. 4E. Spring loaded network model with average NN distances acting as weights in between nodes. Four example simulation outcomes with shuffled weights as input are shown, 1000 iterations (simulation steps) each. We defined a measure of separation as the ratio of the Euclidean distance between the grid node and center of the nodes of all other cell types (inter distance) over the mean distance between nodes of all other (non-grid) cell types (intra distance). The distance ratios are shown for each of the 4 example simulation outcomes.

(E) Dependence of the result of the spring-loaded network model on the number of iterations (simulation steps). The inter to intra (Grid vs. Rest) ratio and distribution (Mean ± SD) is shown for data and shuffled (permuted) weights across [250, 500, 1000, 5000, 10000] iterations (simulation steps) (1000 simulations each). Data in Fig. 4E and SI Appendix, Fig. S5D are shown with 1000 iterations and results tend to become more extreme for even more iterations.

Nearest neighbour (NN) results - continuing Figure 5

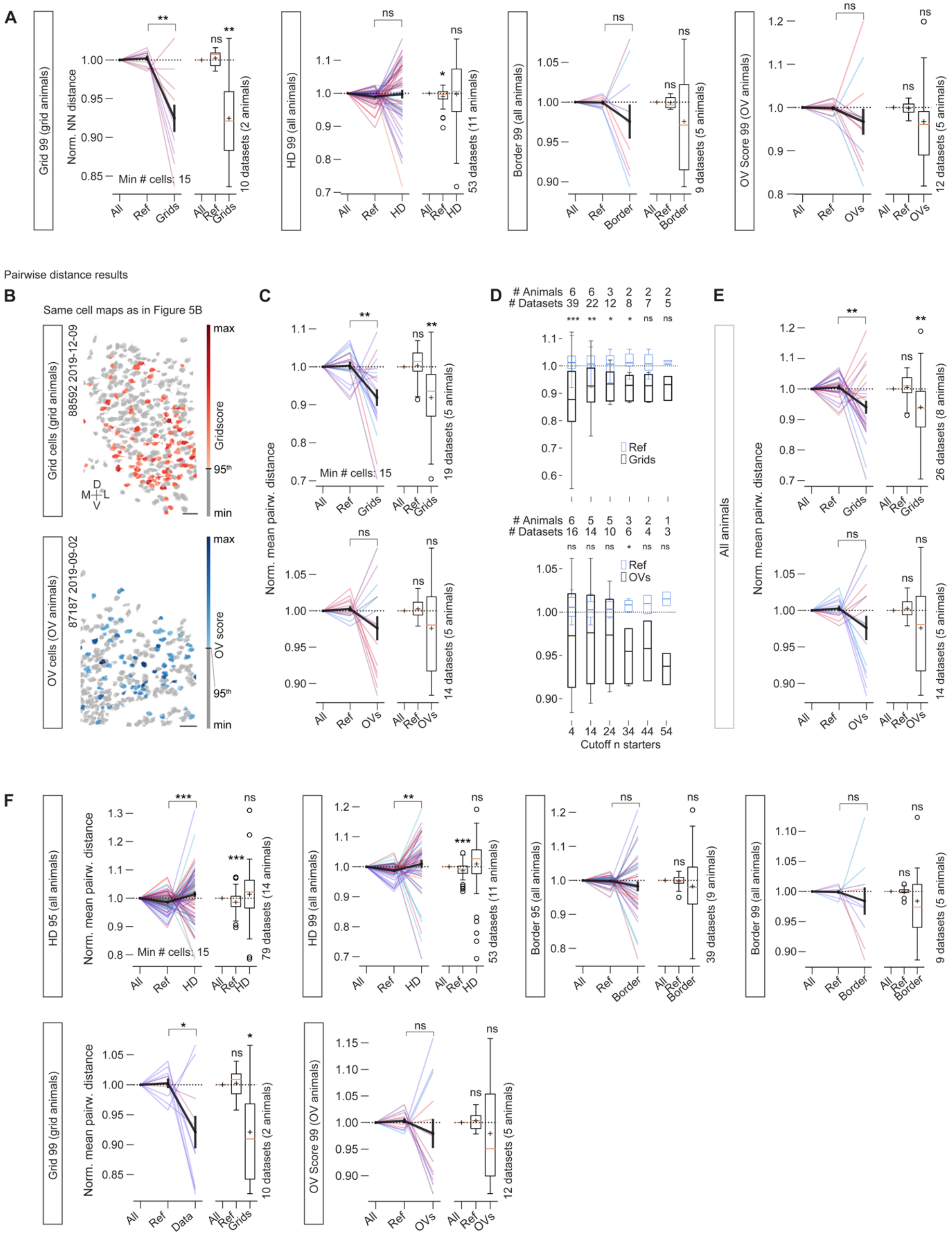

Fig. S6. Extended analysis for clustering of cell classes: NN and pairwise distance analyses.

(A) nearest neighbor (NN) analyses as in Fig. 5 (different cutoffs); **(B)-(F)**: pairwise distance analyses. **(B)**, **(C)** and **(D)** show "Grid animals" on top and "OV animals" on bottom.

(A) Continuation of nearest neighbor (NN) analyses in Fig 5. As for Fig. 5, mean NN distances are normalized to a distribution of cells randomly picked out of all cell locations available for each session and of the same population size as the population of functional cell types under investigation ("All"). A second comparison is shown (reference "Ref"), which consists of cells that were explicitly not part of the cell population under investigation. From left to right: Grid, gridscore cutoff 99th percentile shuffling distribution: Data over 2 "grid" animals, two-sided Mann-Whitney U=89, $n_{Ref}=n_{Grid99}=10$, p=0.004**, Wilcoxon signed-rank test (against 1.): Ref: Z=20, $n_{Ref}=10$, p=0.49 ns Grid99: Z=2 $n_{Grid99}=10$, p=0.0059**; HD, mean vector length cutoff 99th percentile shuffling distribution: data over 11 animals, Mann-Whitney U=1222, $n_{Ref}=n_{HD99}=53$, p=0.25 ns, Wilcoxon signed-rank test (against 1.): Ref: Z=427, $n_{Ref}=53$, p=0.011*, HD99: Z=680 $n_{HD99}=53$, p=0.75 ns;Border, borderscore cutoff 99th percentile shuffling distribution: data over 5 animals, Mann-Whitney U=54, $n_{Ref}=n_{Border99}=9$, p=0.25 ns, Wilcoxon signed-rank test (against 1.): Ref: Z=20, $n_{Ref}=9$, p=0.82 ns, Border99: Z=13, $n_{Border99}=9$, p=0.30 ns; OV, ovscore cutoff 99th percentile shuffling distribution, all other criteria (i.e., information content cutoff and field parameter cutoffs) unchanged: data over 5 "OV" animals, two-sided Mann-Whitney U=105, $n_{Ref}=n_{OV99}=12$, p=0.061 ns, Wilcoxon signed-rank test (against 1.): Ref: Z=35, $n_{Ref}=12$, p= 0.79 ns OV99: Z=24 $n_{OV99}=12$, p=0.27 ns; ns: not significant (p>0.05).

(B) Two sessions (the same as shown in Fig. 5B) in two example animals in which multiple grid cells ("Grid animal", top) or multiple OV cells ("OV animal", bottom) were recorded (color coded by gridscore or OV score). Cells that did not meet cutoff criteria (grid: 95th percentile shuffling) are color coded in grey, animal number and recording date are indicated in top left, anatomical orientation indicated in bottom left (M-medial, L-lateral, V-ventral, D-dorsal). Scale bars 50 µm.

(C) Normalized mean pairwise anatomical distance of grid cells in grid animals (top) and OV cells in object vector animals (bottom). The minimum cutoff for number of functional cells (grid cells in grid animals and OV cells in OV animals) is 15. Pairwise distances are normalized to a distribution of cells randomly picked out of all cell locations available for each session and of the same population size as the population of functional cell types under investigation ("All"). A second comparison is shown (reference "Ref"), which consists of cells that were explicitly not part of the cell population under investigation (i.e., non-grid or non-OV cells; same population size as grid / OV cells). Left: Each line indicates one recording session and colors represent animals. Thick black lines show average and SEM. Grid cells appear to cluster, i.e., pairwise distances are shifted below 1 for grid cells ("Grid" mean ± SEM: 0.92 ± 0.02), while there is a similar trend visible, but no statistical significance, for clustering in OV cells ("OV" mean ± SEM: 0.98 ± 0.02). Box plots show a summary across sessions for data on the left (boxes extend from lower to upper quartile values of the data, with an orange line at the median, whiskers indicate 1st to 99th percentile, outliers are shown as open black circles, black plus signs indicate mean). Vertical labeling towards the right shows the total number of animals and datasets that were used in each comparison. Two-sided Mann-Whitney Grid (top) U=284, $n_{Ref}=n_{Grid}=19$, p=0.0026**; Wilcoxon signed-rank test (against 1.): Ref: Z=79, $n_{Ref}=19$, p=0.54 ns; Grid: Z=20, n_{Grid} =19, p=0.0014**; OV (bottom) Two-sided Mann-Whitney U=121, $n_{Ref}=n_{OV}=14$, p=0.30 ns; Wilcoxon signed-rank test (against 1.): Ref: Z=44, n_{Ref} =14, p=0.63 ns; OV: Z=33, $n_{OV}=14$, p=0.24 ns; ns: not significant (p>0.05).

(D) Comparison as in (C) across different cutoffs (minimum number of functional cells, from 4 to 54) of cells (Grid (top) or OV (bottom)). "Ref" and "Grid"/"OV" summaries are shown as box plots (normalization as in (C)). The number of animals and sessions that remain after filtering are shown above each plot for each comparison. Significance (p < 0.05 *, p < 0.01 **, p < 0.001 ***) indicates results of two-sided Mann-Whitney U test Grid/OV vs. Ref. Boxes extend from lower to upper quartile values of the data, with a thick line at the mean, whiskers indicate 1st to 99th percentile. The observed clustering appears stable over different samples of starter populations in grid cells and trends are visible for OV cells, becoming statistically significant at 34 cells. "Grid animals" mean±SEM for grid cell data and Mann-Whitney U test results: Cutoff 4 cells: 0.88 ± 0.03, U=1225, $n_{Ref}=n_{Grid}=39$, p=3.54e-06***; Cutoff 14 cells: 0.93 ± 0.02, U=367, $n_{Ref}=n_{Grid}=22$, p=0.0035**; Cutoff 24 cells: 0.93 ± 0.02, U=112, $n_{Ref}=n_{Grid}=12$, p=0.023*; Cutoff 34 cells: 0.93 ± 0.02, U=53, $n_{Ref}=n_{Grid}=8$, p=0.031*; Cutoff 44 cells: 0.93 ± 0.02, U=39, n_{Ref} =n_{Grid}=7, p=0.07 ns; Cutoff 54 cells:

0.93 ± 0.03, U=19, n_{Ref} =n_{Grid}=5, p=0.21 ns; "OV animals" mean±SEM for OV cell data and Mann-Whitney U test results: Cutoff 4 cells: 0.97 ± 0.02, U=160, n_{Ref}=n_{OV}=16, p=0.24 ns; Cutoff 14 cells: 0.98 ± 0.02, U=121, n_{Ref}=n_{OV}=14, p=0.30 ns; Cutoff 24 cells: 0.97 ± 0.02, U=64, n_{Ref}=n_{OV}=10, p=0.31 ns; Cutoff 34 cells: 0.95 ± 0.02, U=31, n_{Ref}=n_{OV}=6, p=0.045*; Cutoff 44 cells: 0.96 ± 0.02, U=13, n_{Ref}=n_{OV}=4, p=0.19 ns; Cutoff 54 cells: 0.94 ± 0.02, U=9, n_{Ref}=n_{OV}=3, p=0.08 ns; ns: not significant (p>0.05).

(E) Comparison as in (C) but over all animals and recordings (no preselection for animals, which fall into the categories grid or OV animals). Grid mean ± SEM: 0.94 ± 0.02, Two-sided Mann-Whitney Grid vs. Ref U=496, n_{Ref}=n_{Grid}=26, p=0.004**; Wilcoxon signed-rank test (against 1.): Ref: Z=137, n_{Ref}=26, p=0.33 ns; Grid: Z=74, n_{Grid}=26, p=0.00994**. OV mean ± SEM: 0.98 ± 0.02, Two-sided Mann-Whitney OV vs. Ref U=121, n_{Ref}=n_{OV}=14, p=0.30 ns; Wilcoxon signed-rank test (against 1.): Ref: Z=44, n_{Ref}=14, p=0.63 ns; OV: Z=33, n_{OV}=14, p=0.24 ns. Vertical labeling towards the right shows the total number of animals and datasets that were used in each comparison; ns: not significant (p>0.05).

(F) As in (C) and (E) but for head direction (HD) and border cells (95th or 99th percentile shuffling cutoffs). Compared to grid cells no clean trends are observable for these cell classes.

HD95 mean ± SEM: 1.01 ± 0.01, Two-sided Mann-Whitney HD95 vs. Ref U=2153, n_{Ref}=n_{HD95}=79, p=0.00077***; Wilcoxon signed-rank test (against 1.): Ref: Z=873, n_{Ref}=79, p=0.00055***; HD95: Z=1225, n_{HD}=79, p=0.083 ns; HD99 mean ± SEM: 1.01 ± 0.01, Two-sided Mann-Whitney HD99 vs. Ref U=886, n_{Ref}=n_{HD99}=53, p=0.0011**; Wilcoxon signed-rank test (against 1.): Ref: Z=337, n_{Ref}=53, p=0.00081***; HD: Z=507, n_{HD99}=53, p=0.065 ns; Border95 mean ± SEM: 0.98 ± 0.01, Two-sided Mann-Whitney Border95 vs. Ref U=900, n_{Ref}=$n_{Border95}$=39, p=0.16 ns; Wilcoxon signed-rank test (against 1.): Ref: Z=375, n_{Ref}=39, p=0.83 ns; Border95: Z=287, $n_{Border95}$=39, p=0.15 ns; Border99 mean ± SEM: 0.98 ± 0.02, Two-sided Mann-Whitney Border99 vs. Ref U=48, n_{Ref}=$n_{Border99}$=9, p=0.54 ns; Wilcoxon signed-rank test (against 1.): Ref: Z=20, n_{Ref}=9, p=0.82 ns; Border99: Z=17, $n_{Border99}$=9, p=0.57 ns;

Last row from left to right: Grid cells > 99th percentile cutoff in grid animals. Data over 2 "grid" animals, Mann-Whitney U test Grid vs. Ref: U=78, n_{Ref}=n_{Grid99}=10, p=0.038*; Wilcoxon signed-rank test (against 1.): Ref: Z=24, n_{Ref} =10, p=0.77 ns, Grid: Z=6, n_{Grid99}=10, p=0.027*; OV cutoff 99th percentile shuffling distribution, all other criteria (i.e., information content cutoff and field parameter cutoffs) unchanged: data over 5 "OV" animals, Mann-Whitney U test Grid vs. Ref: U=89, n_{Ref}=n_{OV99}=12, p=0.34 ns; Wilcoxon signed-rank test (against 1.): Ref: Z=32, n_{Ref} =12, p=0.62 ns, OV99: Z=12, n_{OV99}=12, p=0.519 ns; ns: not significant (p>0.05).

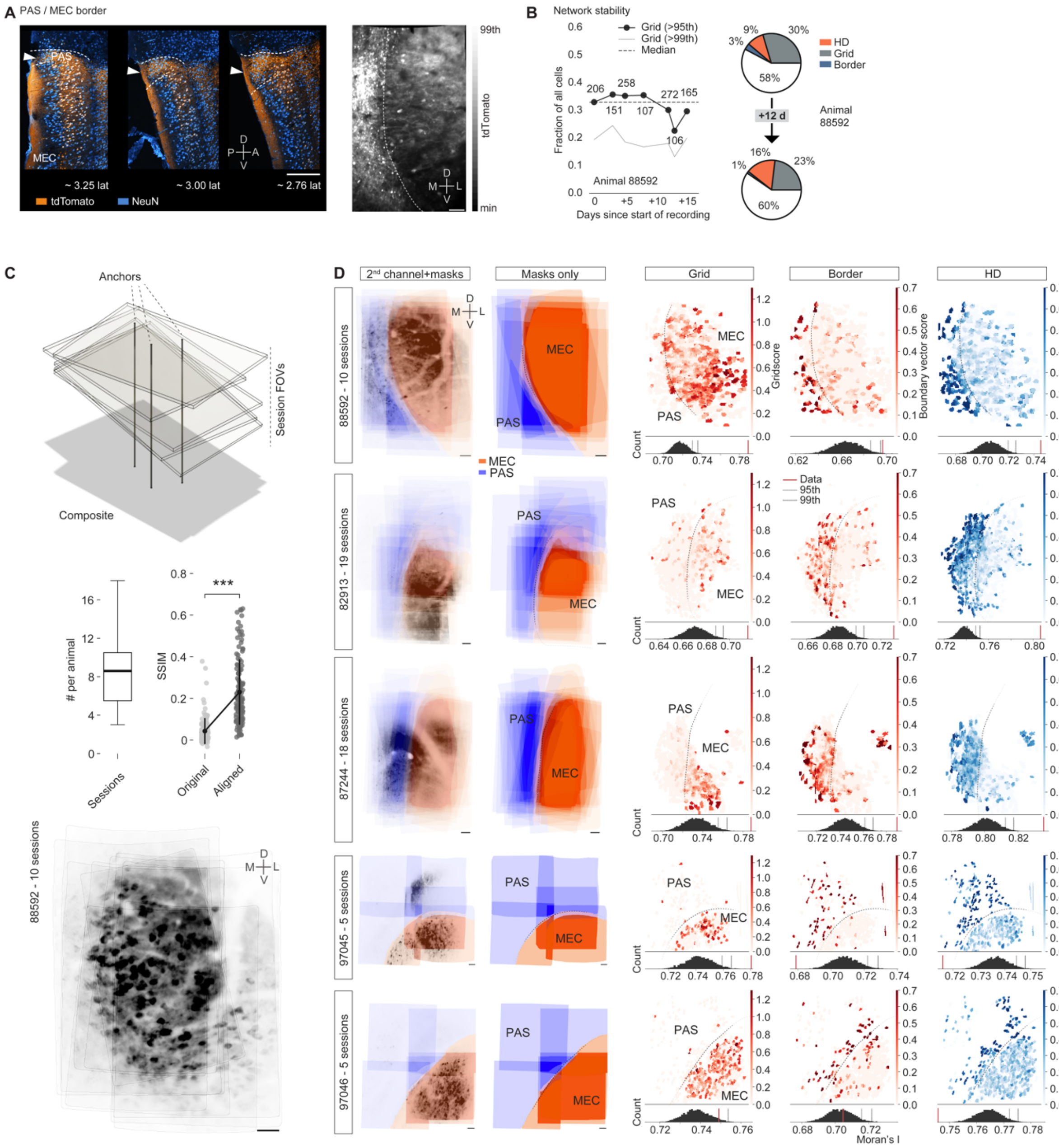

Fig. S7. Stability of functional network composition and identification of PAS/MEC boundary in histology, *in vivo* topographic tuning maps.

(A) Left: Confocal images (maximum intensity projections) of immunostaining in three sagittal brain slices from one animal (cyan - aNeuN, orange - retroAAV tdTomato). Approximate mediolateral position (distance from midline) indicated at the bottom ("lat", unit is mm). Dashed lines indicate the boundary between parasubiculum (PAS) and overlying cortex (top) and the boundary between PAS and medial entorhinal cortex (MEC) (bottom). The boundaries were distinguishable by changes in the pattern of tdTomato labeled neuropil and cellular labeling in MEC. Arrowhead points towards the expanding territory of PAS (lateral to medial); P - posterior, A - anterior, D - dorsal, V - ventral, scale bar 250 µm. Right: Example in vivo imaging field of view (secondary, "red" channel) in the same animal as shown on the left. The boundary between MEC and adjacent PAS is indicated by

a dashed line); M - medial, L - lateral, D - dorsal, V - ventral, scale bar 50 µm; color bar shows range of values (minimum to maximum) in second channel projection.

(B) The composition of functional cell types stayed relatively stable over multiple recorded sessions, here shown for one example animal in which multiple grid cells were co-recorded over many days. Left: Fraction of grid cells meeting either 95th (black line and dots) or 99th percentile (grey line) shuffling cutoffs (dashed line indicates median fraction of cells meeting the 95th percentile cutoff over all sessions). Numbers on top indicate the total number of recorded cells (filtered by SNR). Right: Pie charts showing the fraction of functional cell types in two example sessions of the same animal as on the left. The fractions stay relatively stable.

(C) Top: Schematic of alignment process for multi-session composites. Middle left: Number of sessions per animal, which were aligned (mean=8.6, n=15 animals). Middle right: Structural similarity index measure (SSIM) comparison between overlapping regions in raw ("Original") and aligned image pairs ("Aligned") (Mann-Whitney U=1373, $n_{Original}$=$n_{Aligned}$=134, p=4.20e-33***). Bottom: Composite (average projection) of maximum intensity images for 10 sessions from one animal after alignment. Scale bar 50 µm. **(D)** For every recording, anatomical masks were drawn manually while visualizing the second (red) channel average projection. After stitching recordings (composite FOV based on user defined anatomical landmarks), these masks align to form consistent boundaries across PAS and MEC. Left to right: average projection of stitched and aligned second channel images with anatomical masks overlaid; masks only (anatomical masks are shown with transparency such that the intensity of the color indicates the number of total sessions that were stacked on top of each other); topographic tuning maps as in Fig. 6A, dashed lines indicate approximate location of PAS / MEC (derived from annotations shown on the left. For implant position estimates and coverage of MEC and PAS based on port-mortem histology see SI Appendix, Fig. S2 H; Scale bars 50 µm.

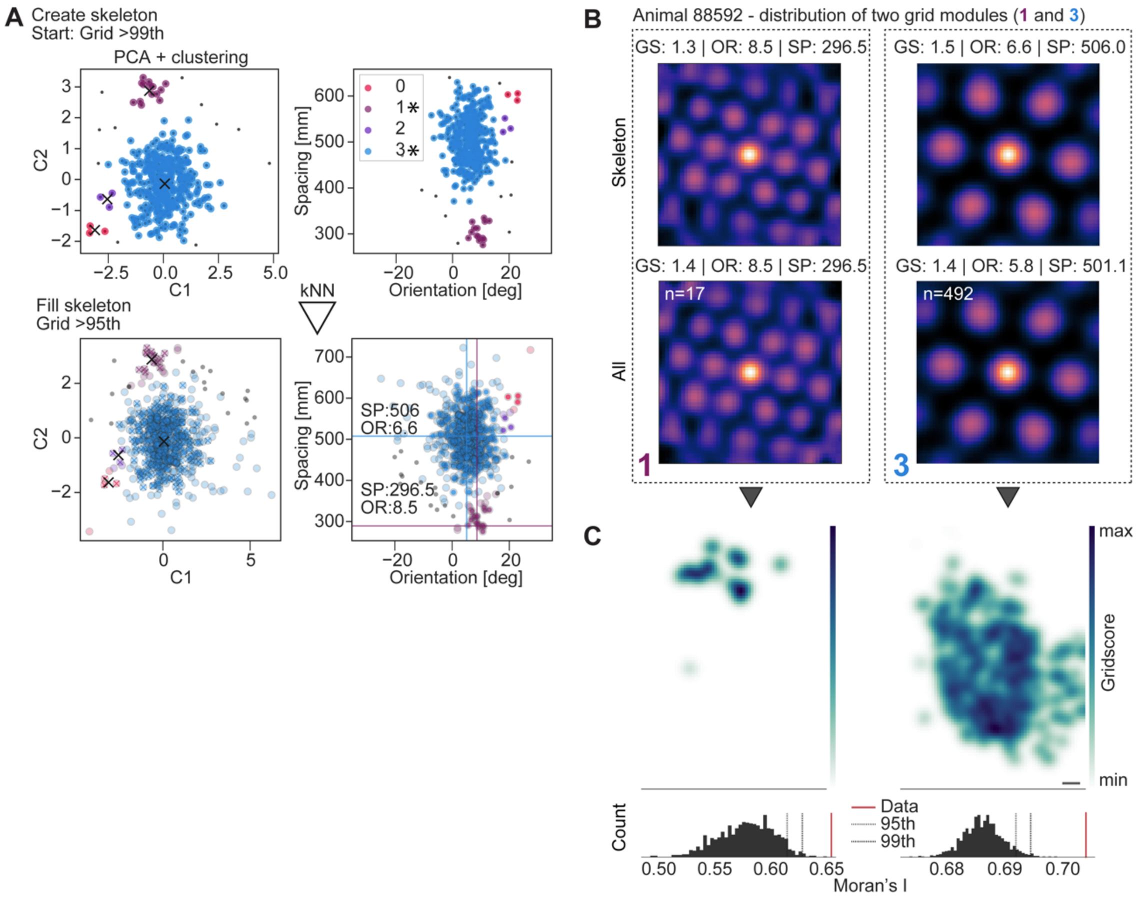

Fig. S8. Grid module extraction and anatomical mapping.

(A) Grid module extraction. Example data from one animal with many grid cells. After dimensionality reduction (PCA, left, C1 and C2 - principal components 1 and 2), unsupervised clustering is run on a core set of "pure" grid cells (>99th percentile cutoff) to extract seed clusters ("skeleton", top). A k-nearest neighbor classifier (kNN) is trained on this data; this classifier is subsequently used to expand each module by adding grid cells with >95th (and <99th) percentile shuffling cutoff (bottom). Four candidate modules were extracted / expanded for this example animal. The two main modules (those with high numbers of classified cells) – modules 1 (spacing ~ 297 mm and orientation ~ 9 degrees, magenta dots, lines indicate means) and 3 (spacing ~ 506 mm and orientation ~ 7 degrees, cyan dots, lines indicate means) – are shown in (B) and (C).

(B) Average spatial autocorrelation maps of modules 1 and 3 (extracted in (A)). Top: Module properties of a subset of "pure" grid cells (> 99th percentile shuffling cutoff) used as "Skeleton" and all cells on bottom ("All") (GS - Grid Score, OR - orientation [degrees], SP - spacing [mm], total number of cells (n=17 for smaller (left, module 1) and n=492 for larger (right, module 3) module)).

(C) Modules extracted in (A) and (B), mapped over anatomical space (Left: Module 1, Right: Module 3 over the same FOVs). Topographical tuning maps, Moran's I distributions and cutoffs (bottom) as in Fig. 6A and SI Appendix, Fig. S7D. Colorbar shows grid tuning mapped from minimum to maximum for the scoremap average projection (7 sessions). Scale bar: 50 µm.

Extended References

1. J. B. Wekselblatt, E. D. Flister, D. M. Piscopo, C. M. Niell, Large-scale imaging of cortical dynamics during sensory perception and behavior. *J. Neurophysiol.* **115**, 2852–2866 (2016).

2. FELASA working group on revision of guidelines for health monitoring of rodents and rabbits, *et al.*, FELASA recommendations for the health monitoring of mouse, rat, hamster, guinea pig and rabbit colonies in breeding and experimental units. *Lab. Anim.* **48**, 178–192 (2014).

3. D. G. R. Tervo, *et al.*, A Designer AAV Variant Permits Efficient Retrograde Access to Projection Neurons. *Neuron* **92**, 372–382 (2016).

4. Q. Wang, *et al.*, The Allen Mouse Brain Common Coordinate Framework: A 3D Reference Atlas. *Cell* **0** (2020).

5. A. L. Tyson, *et al.*, Tools for accurate post hoc determination of marker location within whole-brain microscopy images. *bioRxiv*, 2021.05.21.445133 (2021).

6. W. Zong, *et al.*, Fast high-resolution miniature two-photon microscopy for brain imaging in freely behaving mice. *Nat. Methods* (2017) https:/doi.org/10.1038/nmeth.4305.

7. W. Zong, *et al.*, Miniature two-photon microscopy for enlarged field-of-view, multi-plane and long-term brain imaging. *Nat. Methods* **18**, 46–49 (2021).

8. W. Zong, *et al.*, Large-scale two-photon calcium imaging in freely moving mice. *bioRxiv*, 2021.09.20.461015 (2021).

9. T. A. Pologruto, B. L. Sabatini, K. Svoboda, ScanImage: flexible software for operating laser scanning microscopes. *Biomed. Eng. Online* **2**, 13 (2003).

10. D. Yatsenko, *et al.*, DataJoint: managing big scientific data using MATLAB or Python. *bioRxiv* (2015) https:/doi.org/10.1101/031658.

11. Ø. A. Høydal, E. R. Skytøen, S. O. Andersson, M.-B. Moser, E. I. Moser, Object-vector coding in the medial entorhinal cortex. *Nature* (2019) https:/doi.org/10.1038/s41586-019-1077-7.

12. M. Pachitariu, *et al.*, Suite2p: beyond 10,000 neurons with standard two-photon microscopy. *bioRxiv*, 061507 (2017).

13. C. Stringer, M. Michaelos, M. Pachitariu, Cellpose: a generalist algorithm for cellular segmentation. *bioRxiv*, 2020.02.02.931238 (2020).

14. J. Friedrich, P. Zhou, L. Paninski, Fast online deconvolution of calcium imaging data. *PLoS Comput. Biol.* **13**, e1005423 (2017).

15. E. van der Velden, CMasher: Scientific colormaps for making accessible, informative and "cmashing" plots. *J. Open Source Softw.* **5**, 2004 (2020).

16. K. Barbary, SEP: Source Extractor as a library. *J. Open Source Softw.* **1**, 58 (2016).

17. P. Berens, CircStat: A MATLAB Toolbox for Circular Statistics. *J. Stat. Softw.* **31**, 1–21 (2009).

18. D. C. Rowland, *et al.*, Functional properties of stellate cells in medial entorhinal cortex layer II. *Elife* **7** (2018).

19. J. H. Zar, *Biostatistical Analysis* (Prentice Hall, 2010).

20. T. Solstad, C. N. Boccara, E. Kropff, M.-B. Moser, E. I. Moser, Representation of Geometric Borders in the Entorhinal Cortex. *Science* **322**, 1865–1868 (2008).

21. W. Skaggs, B. McNaughton, K. Gothard, An Information-Theoretic Approach to Deciphering the Hippocampal Code in *Advances in Neural Information Processing Systems*, S. Hanson, J. Cowan, C. Giles, Eds. (Morgan-Kaufmann, 1993).

22. F. Pedregosa, *et al.*, Scikit-learn: Machine Learning in Python. *J. Mach. Learn. Res.* **12**, 2825–2830 (2011).

23. Z. Wang, A. C. Bovik, H. R. Sheikh, E. P. Simoncelli, Image quality assessment: from error visibility to structural similarity. *IEEE Trans. Image Process.* **13**, 600–612 (2004).

24. G. Cho, Spatial Processes: Models and Applications by A.D. Cliff and J.K. Ord. 16 by 24 em, 266 pages, maps, diags., index and bibliography. london: Pion Limited, 1981. (ISBN 08-85086-081-4). £20.50. *Cartography* **13**, 59–60 (1983).

25. S. J. Rey, L. Anselin, “PySAL: A Python Library of Spatial Analytical Methods” in *Handbook of Applied Spatial Analysis: Software Tools, Methods and Applications*, M. M. Fischer, A. Getis, Eds. (Springer Berlin Heidelberg, 2010), pp. 175–193.

26. P. Virtanen, *et al.*, SciPy 1.0: fundamental algorithms for scientific computing in Python. *Nat. Methods* **17**, 261–272 (2020).
